# Disruption of white matter connectivity in chronic obstructive pulmonary disease

**DOI:** 10.1371/journal.pone.0223297

**Published:** 2019-10-03

**Authors:** Catherine A. Spilling, Paul W. Jones, James W. Dodd, Thomas R. Barrick

**Affiliations:** 1 Neuroscience Research Centre, Molecular and Clinical Sciences Research Institute, St George’s University of London, Tooting, London, United Kingdom; 2 Institute of Infection and Immunity, St George's, University of London, Tooting, London, United Kingdom; 3 Academic Respiratory Unit, Second Floor, Learning and Research, Southmead Hospital, University of Bristol, Westbury-on-Trym, Bristol, United Kingdom; University at Buffalo, UNITED STATES

## Abstract

**Background:**

Mild cognitive impairment is a common systemic manifestation of chronic obstructive pulmonary disease (COPD). However, its pathophysiological origins are not understood. Since, cognitive function relies on efficient communication between distributed cortical and subcortical regions, we investigated whether people with COPD have disruption in white matter connectivity.

**Methods:**

Structural networks were constructed for 30 COPD patients (aged 54–84 years, 57% male, FEV_1_ 52.5% pred.) and 23 controls (aged 51–81 years, 48% Male). Networks comprised 90 grey matter regions (nodes) interconnected by white mater fibre tracts traced using deterministic tractography (edges). Edges were weighted by the number of streamlines adjusted for a) streamline length and b) end-node volume. White matter connectivity was quantified using global and nodal graph metrics which characterised the networks connection density, connection strength, segregation, integration, nodal influence and *small-worldness*. Between-group differences in white matter connectivity and within-group associations with cognitive function and disease severity were tested.

**Results:**

COPD patients’ brain networks had significantly lower global connection strength (*p* = 0.03) and connection density (*p* = 0.04). There was a trend towards COPD patients having a reduction in nodal connection density and connection strength across the majority of network nodes but this only reached significance for connection density in the right superior temporal gyrus (*p* = 0.02) and did not survive correction for end-node volume. There were no other significant global or nodal network differences or within-group associations with disease severity or cognitive function.

**Conclusion:**

COPD brain networks show evidence of damage compared to controls with a reduced number and strength of connections. This loss of connectivity was not sufficient to disrupt the overall efficiency of network organisation, suggesting that it has redundant capacity that makes it resilient to damage, which may explain why cognitive dysfunction is not severe. This might also explain why no direct relationships could be found with cognitive measures. Smoking and hypertension are known to have deleterious effects on the brain. These confounding effects could not be excluded.

## Introduction

Mild cognitive impairment is a relatively common feature of a number of chronic diseases, including diabetes, kidney disease and rheumatoid arthritis [1–3]. Chronic obstructive pulmonary disease (COPD) is a chronic respiratory disease which is one of the leading causes of morbidity and mortality worldwide. It is associated with a number of extra-pulmonary co-morbid conditions, which occur more frequently in COPD than in smokers or never smokers, suggesting an intrinsic link to the disease [4]. One such co-morbidity is cognitive dysfunction, with estimates of its prevalence ranging from 10–61% [5]. Whilst the deficit is not universally very severe, its presence is associated with greater disability [6], poorer medication compliance [7] and an elevated risk of an exacerbation of their respiratory symptoms and mortality [6]. The pathophysiological origins of this cognitive dysfunction are not understood, but may involve structural and functional changes to brain anatomy secondary to cerebral small-vessel disease (SVD) [8–11]. Diffusion tensor imaging studies have reported a diffuse pattern of diffusion abnormalities in COPD suggestive of widespread deterioration of the tissue microstructure [10,12,13]. In SVD comparable diffusion abnormalities have been found to correlate more strongly with cognitive function and better predict cognitive decline and conversion to dementia [14] than conventional markers of SVD [15,16].

Cognitive function is reliant on efficient communication between networks of distributed brain regions interconnected by white matter fibre tracts [17,18]. This network complexity manifests as a hierarchical modular organisation (i.e. highly integrated sub-networks nested within larger networks) [19] featuring both global and nodal ‘small-world’ properties and an exponentially truncated power law degree-distribution indicative of the presence of a small number of heavily connected ‘hub’ brain regions [20]. It follows that pathology which disconnects white matter fibres or perturbs the network configuration, will be deleterious to function [17,18]. Indeed, it has been reported that the relationship between diffusion abnormalities and cognitive dysfunction in SVD is mediated by structural network disruption [21]. It is plausible that the same process is responsible for cognitive dysfunction in COPD.

The present study provides an exploratory cross-sectional patient-control investigation of large-scale structural networks in a well-defined cohort of stable patients with COPD. We hypothesised that patients with COPD would have impaired white matter connectivity relative to control subjects and that the magnitude of this network disruption would be related to lower cognitive function and greater disease severity.

## Materials and methods

### Subjects

31 stable COPD patients were recruited as part of a previous study [10]. Data from six of these patients were unavailable at the time of the original publication [10]. All participants were outpatients recruited from St George’s University Hospital and Royal Brompton Hospital between 2010 and 2011, 17 of whom, had not been hospitalised within the preceding 12 months of data collection. The remaining 14 had previously been inpatients admitted to St. George’s Hospital NHS Trust with a primary diagnosis of COPD exacerbation from whom data were obtained within 12 months of discharge. All participants were assessed whilst in a stable condition. Diffusion data was unavailable for one patient. Additionally, 26 non-COPD control subjects were recruited from the local community, three of whom were later excluded, two due to a scanner fault and one due to the presence of additional neuropathology (see [10] for a full list of exclusion criteria). This resulted in a cohort of 30 COPD patients and 23 controls. All participants provided written informed consent. This study was approved by Wandsworth and East Central London Research Ethics Committees (Ref: 10/H0721/16) and by St George’s University of London, Joint Research Office (Ref: 090147).

Demographic and clinical characteristics of this cohort can be viewed in Table 1. To summarise, patients with COPD were well-matched for age and sex (aged 54–84 years, 57% male) compared to controls (aged 51–81 years, 48% Male). Patients with COPD met the Global Initiative for Chronic Obstructive Lung Disease (GOLD) classification [22] for moderate-severe airflow obstruction (FEV_1_ = 52.5 ± 21.1% pred., GOLD stage 3 (1), median (IQR)) and were not significantly hypoxaemic (PO_2_ = 9.9 ± 2.5 KPa) and were normocapnic (PCO_2_ = 5.0 ± 0.7 kPa). Patients with COPD had smoked for a significantly greater number of pack years and were significantly more anxious and depressed than controls (see Table 1). Only one COPD patient met the Mini Mental State Examination (MMSE) criteria for severe cognitive impairment, however, patients with COPD had significantly lower estimated pre-morbid IQ and lower cognitive function across all the cognitive domains assessed (see Table 1).

**Table 1:** Demographics.

	Controls	COPD	Statistic *(df)*	*p*
N	23	30		
Age	65.6 ± 7.4	67.2 ± 8.3	0.760 (51)	0.451^1^
Males (%)	47.8	56.7	1.385	0.665^2^
Height (m)	1.7 ± 0.1	1.7 ± 0.1	-0.570 (45)	0.572^1^
Body mass index (kg/m^2^)	26.9 ± 4.7	26.6 ± 4.4	-0.266 (43)	0.792^1^
Smoking (pack years)	0.0 (4.0)	53.5 (27.0)	682.5	<0.0001^3^****
Cardiovascular risk (FSRP)	6.1 ± 3.2	7.2 ± 4.1	1.046 (51)	0.301^1^
Exacerbations in last 12 months	-	1.0 (3.0)	-	-
SGRQ–total (health status)	-	53.7 ± 30.0	-	-
SGRQ—symptoms	-	62.9 ± 21.7	-	-
SGRQ—activity	-	73.2 (22.6)	-	-
SGRQ—impacts	-	41.8 ± 18.9	-	-
Co-morbidity Index	0 (0)	0 (1)	232.0	0.009^3^**
HADS–anxiety	3.9 ± 2.8	7.4 ± 4.5	3.390 (44.4)	0.002^1^^a^**
HADS–depression	1 (4)	5 (7)	3.061	0.002^3^**
HADS–total	6.8 ± 5.0	11.8 ± 8.0	2.788 (49.4)	0.008^1^^a^**
Cognitive Function				
Estimated pre-morbid IQ	110.0 (16.0)	103.0 (16.8)	-2.552	0.011^3^*
Executive function	12.3 ± 2.6	9.4 ± 2.5	-4.096 (51)	<0.001^1^***
Episodic memory	10.9 ± 3.1	9.3 ± 2.4	-2.147 (51)	0.037^1^*
Processing speed	108.0 (18.0)	89.5 (24.8)	178.5	0.002^3^**
Working memory	106.6 ± 15.5	94.2 ± 12.5	3.229 (51)	0.002^1^**
MMSE	30.0 (1.0)	28.0 (2.0)	154.5	<0.001^3^***
Lung Function				
FEV_1_ (% pred.)	-	52.5 ± 21.1	-	-
FVC (% pred.)	-	86.0 ± 32.1	-	-
FEV_1_/FVC (%)	-	48.9 ± 15.8	-	-
GOLD Stage I (%)	-	10	-	-
GOLD Stage II (%)	-	31	-	-
GOLD Stage III (%)	-	35	-	-
GOLD Stage IV (%)	-	17	-	-
Normal FEV_1_/FVC at assessment (%)	-	7	-	-
Arterial Blood Gases				
PO_2_ (kPa)	-	9.9 (2.5)	-	-
PCO_2_ (kPa)	-	5.0 (0.7)	-	-
pH	-	7.4 ± 0.0	-	-

Group comparison of demographic and clinical characteristics for the COPD patient group (aged 54–84 years, 57% male) and control group (aged 51–81 years, 48% Male). For Gaussian data,

^1^independent t-tests, group means ± standard deviations, *t*-statistics, degrees of freedom (df) and *p*-values (*p*) are reported. For categorical data,

^2^chi-squared tests, group percentages, chi-square statistics and *p*-values (*p*) are reported. For non-Gaussian data,

^3^Mann-Whitney U tests, group medians (interquartile ranges), *U* statistics and exact probabilities (*p*) are reported.

^a^Correction for unequal variances.

Significant at *p<0.05,

**p<0.01,

***p<0.001 and

****p<0.0001.

### Statistical power

The sample size was informed by past empirical evidence and scientific reasoning. A sample size of N = 55 was chosen based on feasibility, economic grounds and previous research by other authors [11,23]. No formal power calculation was performed.

### Cognitive and disease severity measures

Full details have been provided previously [10]. To summarise, post-bronchodilator spirometry, arterial blood gas analysis, a modified form of the Framingham Stroke Risk Profile (FSRP) [10,24], Charlson Co-morbidity Index [25] and a health status measure–the St George’s Respiratory Questionnaire (SGRQ) [26] were administered to the patient group only. These measures were not collected for the controls as they were healthy individuals, therefore, we could not formally confirm that controls had normal lung function and blood gases. All subjects completed the Hospital Anxiety and Depression Scale (HADS) [27] and neuropsychological assessment, including the Mini Mental State Examination (MMSE), the Wechsler Test of Adult Reading (providing an estimate of pre-morbid IQ) and sub-scales taken from the Wechsler Adult Intelligence Scale–III, the Wechsler Memory Scale–III, the Delis-Kaplan Executive Function System, and the Rey-Complex Figure Test and Recognition Trial (see [10] for the specific subtests used). Composite scores were calculated, assessing the following cognitive domains: Executive Function (average of the Delis-Kaplan Executive Function System scaled scores), Episodic Memory (combined average of Wechsler Memory Scale–III and Rey-Complex Figure Test and Recognition Trial scaled scores), Processing Speed (Processing Speed Index from the Wechsler Adult Intelligence Scale–III) and Working Memory (Working Memory Index from the Wechsler Adult Intelligence Scale–III) [10].

### Image acquisition and pre-processing

Magnetic resonance (MR) images were obtained for all subjects, using a 3-Tesla Philips Achieva dual TX scanner equipped with a 32-channel head coil and gradients up to a maximum of 80 mT/m, at St George’s University of London. T1-weighted 3D volume images were acquired using a Turbo Field Echo sequence (TE = 3700ms, TR = 8200ms, flip angle = 8°, providing 160 contiguous sagittal slices with an isotropic voxel dimension of 1mm^3^ and field-of-view (FOV) of 240x240mm^2^). Fluid Attenuated Inversion Recovery images (FLAIR) were acquired using an inversion recovery sequence (TE = 125 ms, TR = 11000 ms, TI = 2800 ms with 60 contiguous axial slices of 3 mm slice thickness, FOV = 240 × 240 mm^2^ and voxel dimension 0.96^2^x3mm^3^). Diffusion-weighted images (DWI) were acquired using a diffusion sensitised, single-shot spin-echo planar sequence (TE = 75ms, TR = 6450ms, 60 contiguous axial slices, FOV = 224x224mm^2^ in a 112x112 matrix and voxel dimension 2mm^3^). The first eight DWI volumes were acquired without diffusion sensitisation (b = 0s mm^-2^). The remaining, were obtained with diffusion gradients applied in 32 non-collinear directions (b = 1000s mm^-2^). DWI were simultaneously corrected for the geometric distortions caused by eddy currents and movement artefacts using FSL’s ‘eddy-correct’ (FSL, version 5.0.6, FMRIB, Oxford, http://fsl.fmrib.ox.ac.uk/fsl/fslwiki/). Diffusion tensors were computed at every voxel within the DWI using FSL’s ‘dtifit’ [28] and the skull removed using FSL’s ‘BET’ [29]. Fractional Anisotropy (FA) was calculated at every voxel within the diffusion tensor images (DTI), representing the local ‘directionality’ of diffusion.

#### Total intracranial volume and white matter hyperintensities

Supratentorial grey matter, white matter and CSF tissues were segmented from the T1-weighted images, and white matter hyperintensities of presumed vascular origin (WMHs) were segmented from the combined tissue intensities from the T1-weighted and FLAIR using a semi-automated procedure adapted from the standard SPM pipeline (SPM version 12, 2014, https://www.fil.ion.ucl.ac.uk/spm/). This is described in full in [11,30]. Tissue volumes were quantified by integrating tissue pixel volume contributions within each segmentation, and total intracranial volume was calculated as the sum of grey matter, white matter and CSF volumes. WMH volumes were presented as a percentage of total intracranial volume [11].

#### Network construction

**Fig 1:**
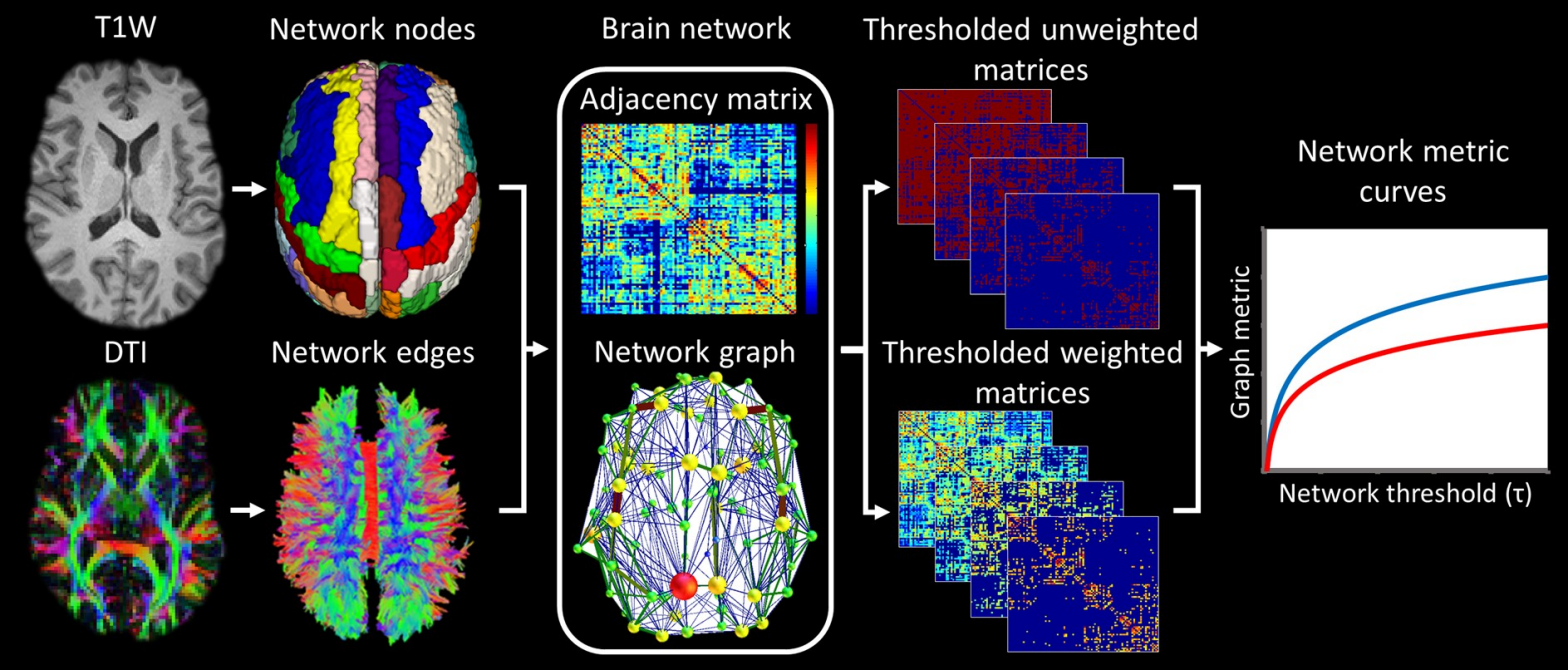
Network construction. The native T1-weighted images were co-registered to the DTI and transformed to Montreal Nerological Institute space. These transforms were combined, inverted and applied to the AAL atlas (excluding cerebellum) parcellating 90 anatomical regions on the DTI (Network nodes). White matter fibre tracts were traced from the DTI (Network edges). Structural networks were defined from the nodes and edges, and the edges weighted by the number of constituent streamlines adjusted for streamline length and end-node volume. Networks were thresholded across 40 edge weighting and edge density thresholds. Weighted and unweighted network metrics were calculated at each threshold and used to construct network metric curves.

Brain networks can be regarded as a graph comprising a set of nodes interconnected by a set of edges [31]. In the present study, structural networks were constructed using the workflow shown in Fig 1. Network nodes consisted of 90 anatomical grey matter regions defined using the Automated Anatomical Labelling atlas [32] and network edges consisted of the white matter fibre tracts interconnecting these grey matter regions (traced using deterministic tractography).

Node definition: Native T1-weighted images were co-registered to the b0 in native DTI-space using boundary-based registration using FSL’s (FSL version 5.0.6) ‘epi-reg’ script [33]. The T1-weighted images were normalised to the high resolution T1-weighted Montreal Neurological Institute (MNI) template image [34] provided with MRIcro (MRIcro, version 6, 2013, www.mricro.com) using a symmetric diffeomorphic non-linear transformation applied via Advanced Normalization Tools [35] (ANTs, version 1.9, http://stnava.github.io/ANTs/). These two transformations were combined, inverted and applied to the Automated Anatomical Labelling atlas (excluding the cerebellum) [32] thereby parcellating the native DTI into 90 supratentorial cortical and subcortical grey matter regions. Each region represented a node of the network.

Edge definition: The trajectory of white matter fibre bundles was reconstructed from the DTI using an in-house deterministic tractography algorithm. The algorithm was seeded across the whole-brain using a super-resolution grid (0.5 mm^3^) with a step-size of 0.5 mm [36]. Streamlines were terminated if FA dropped below 0.2 or if the angle between principal eigenvectors exceeded 40°. Edges were defined as the presence or absence of a streamline interconnecting node pairs. This resulted in a 90 x 90 adjacency matrix. Weights were ascribed to edges using two weighting strategies with higher weightings given to edges composed of a greater number of tractography streamlines. The two strategies included different corrections for biases inherent to network construction.

Streamline length adjusted weighting strategy: Network weights (*w*_*ij*_^*lngth*^) were defined according to a modified form [21] of Hagmann et al’s [37] formula, where *l* is the length (mm) of the set of *N* unique streamlines terminating in nodes *i* and *j*,

$$w_{ij}^{lngth}=\frac{1}{2}\overset{}{\underset{}{\sum_{m=0}^{N}}}\frac{1}{l_{m}}.$$ (1)

This method corrects for the distal bias caused by the relationship between length of tractography streamlines and their constituent number (due to longer streamlines containing a greater number of tractography seed points).

Volume-adjusted weighting strategy: Weights (*w*_*ij*_^*vol*^) were defined using a modified form of Heuvel and Sporns equation [38], where the number of streamlines terminating in nodes *i* and *j* was normalised by the sum of end-node volumes *V*,

$$w_{ij}^{vol}=\frac{2N}{\left(V_{i}+V_{j}\right)}.$$ (2)

Larger node volumes contain a greater number of tractography seed points and will therefore have a higher probability of being connected by streamlines. This confound was adjusted for by normalising the number of streamlines by the combined end-node volumes. This method also corrects for localised atrophy of grey matter nodes and for differences in head size.

#### Summary network metrics

Summary network graph metrics were selected that could be calculated for both weighted and unweighted networks. These described the nodal and global topology of the networks in terms of edge density (unweighted degree), streamline density (weighted degree also known as *strength*), segregation (unweighted and weighted local efficiency), integration (unweighted and weighted nodal efficiency) and nodal influence (unweighted and weighted betweenness centrality). These were computed at each network node using the brain connectivity toolbox (http://www.brain-connectivity-toolbox.net/) [39] providing nodal network metrics and averaged across all nodes to provide global network metrics (metric definitions and details can be found in Table 2). Additionally, weighted and unweighted *small-worldness* were quantified—composite measures calculated from the characteristic path length and clustering coefficient normalised by the equivalent metrics computed over 100 randomly re-wired networks [40]. Networks were considered to have ‘small-world’ topological properties when unweighted or weighted *small-worldness* >>1.

**Table 2:** *Network metric definitions*.

	Unweighted metrics	Weighted metrics
Edge and connection density		
Degree (*k*)	$k_{i}=\sum_{j\in N}a_{ij},$ where *N* is the set of all nodes in the network, (*i*,*j*) is an edge connecting nodes *i* and *j*, and *a*_*ij*_ is the edge connection status between nodes *i* and *j* i.e. present = 1, absent = 0.	$k_{i}^{w}=\sum_{j\in N}w_{ij},$ where *w*_*ij*_ is the normalised edge weight 0 ≤ w_*ij*_ ≤ 1, where normalisation has been performed by dividing weights by the maximum weight in the network.
Segregation		
Local Efficiency (*E*_*loc*_)	$E_{loc,i}=\frac{1}{n}\sum_{i\in N}\frac{\sum_{j,h\in N,j\neq i}a_{ij}a_{ij}{[d}_{jh}(N_{i}){]}^{-1}}{k_{i}(k_{i}-1)},$ where is the *d*_*jh*_*(N*_*i*_*)* is the length of the shortest path between *j* and *h*, that contains only neighbours of *i*.	$E_{loc,i}^{w}=\frac{1}{2}\sum_{i\in N}\frac{\sum_{j,h\in N,j\neq i}{\left(w_{ij}w_{ij}[d_{jh}^{w}\left(N_{i}\right){]}^{-1}\right)}^{\frac{1}{3}}}{k_{i}(k_{i}-1)},$
Clustering Coefficient (*C*)	$C_{i}=\frac{1}{n}\sum_{i\in N}\frac{{2t}_{i}}{k_{i}(k_{i}-1)},$ where n is the number of nodes in the network and is the *t*_*i*_ number of triangles around node *i*, $t_{i}=\sum_{j,h\in N}a_{ij}a_{ih}a_{jh},$	$C_{i}^{w}=\frac{1}{n}\sum_{i\in N}\frac{{2t}_{i}^{w}}{k_{i}(k_{i}-1)},$ where $t_{i}^{w}=\sum_{j,h\in N}a_{ij}a_{ih}a_{jh},$
Integration		
Global Efficiency (*E*)	$E_{i}=\frac{1}{n}\sum_{i\in N}\frac{\sum_{j\in N,j\neq i}d_{ij}^{-1}}{n-1}$, where *d*_*ij*_ is the distance or length of the shortest path between nodes *i* and *j*, defined as the number of edges forming the shortest topological route between nodes *i* and *j*.	$E_{i}^{w}=\frac{1}{n}\sum_{i\in N}\frac{\sum_{j\in N,j\neq i}{{(d}_{ij}^{w})}^{-1}}{n-1}$, where *d*_*ij*_^*w*^ is the distance or length of the shortest path between nodes *i* and *j*, defined as the sum of the inverse of weights forming the shortest topological route between nodes *i* and *j*.
Characteristic Path Length (*L*)	$L_{i}=\frac{1}{n}\sum_{i\in N}\frac{\sum_{j\in N,j\neq i}d_{ij}}{n-1},$	$L_{i}^{w}=\frac{1}{n}\sum_{i\in N}\frac{\sum_{j\in N,j\neq i}d_{ij}^{w}}{n-1}$,
Nodal influence		
Betweenness Centrality (*b*)	bi=1(n-1)(n-1)∑h,j∈Nh≠j,h≠i,jρhj(i)ρhj, where *ρ*_*hj*_ is the number of shortest paths between nodes *h* and *j*, and *ρ*_*hj*_^(i)^ is the number of shortest paths that pass through node *i*.	bijw=1(n-1)(n-1)∑h,j∈Nh≠j,h≠i,jρhj(i)ρhj,
Small-world structure		
Small-worldness (*S*)	$S=\frac{C/C_{rand}}{L/L_{rand}},$ where *C*_*rand*_ and *L*_*rand*_ are the average unweighted clustering coefficient and average unweighted characteristic path length computed on 100 randomly re-wired networks.	$S^{w}=\frac{C^{w}/C_{rand}^{w}}{L^{w}/L_{rand}^{w}}$, where C^W^_*rand*_ and *L*^*W*^_*rand*_ are the average weighted clustering coefficient and average weighted characteristic path length computed on 100 randomly re-wired networks.

Adapted from [39].

#### Network thresholding

The networks were thresholded so that low weight network edges that are likely to have been generated by noise within the DTI data, were removed. The choice of threshold is largely arbitrary [41]. Additionally, the topological properties of a network are highly dependent on the number of edges in the network, therefore, it is necessary to control for edge density when evaluating network topology [42]. Consequently, all network metrics were assessed at 40 evenly-spaced fixed levels of edge density except for unweighted degree, itself an indicator of edge density, which was assessed across 40 fixed levels of edge weighting. The upper edge density threshold was determined by the maximum value at which all subjects’ networks could be successfully density-matched. Edge weighting thresholds were set as the average edge weight for the average subject at each density threshold.

Network metric curves were constructed by plotting the network metric value at each network threshold. At high edge weighting thresholds and low edge density thresholds, low weight edges will have been removed, meaning that hub nodes will have a greater influence over the network metrics at these thresholds.

### Statistical analysis

Between-group differences in WMHs and total intracranial volume were tested using ANCOVAs. WMHs were log_10_-transformed to correct for non-Gaussianity prior to analysis. Between-group differences in nodal and global network graph metrics were tested for the total area under each metric curve (AUC_total_) and at every point along the metric curve (*point-by-point)*. This latter method was used to verify the AUC_total_ results and to determine which network thresholds were primarily contributing to significant effects. For the AUC_total_ method, between-group comparisons were performed using parametric (Gaussian data and data that could be log_10_-transformed to Gaussian) and non-parametric permutation ANCOVAs with 10000 permutations (non-Gaussian data), performed using SPSS version 24 (IBM Corp, 2015) and FSL’s randomise (FSL version 5.0.6) (Winkler *et al*., 2014), respectively. Residuals were checked for gaussianity using histograms and quantile-quantile plots. Results were Bonferroni corrected for multiple comparisons. Statistical testing for the *point-by-point* analyses was performed using permutation ANCOVAs and corrected for multiplicity across the network thresholds using the multi-threshold permutation correction (MTPC) method [43,44] performed using in-house software. For results to be considered significant they were required to exceed the familywise error (FWE) adjusted critical threshold and the area under the curve (AUC_MTPC_) of supra-critical clusters of results had to exceed the average AUC_MTPC_ of supra-critical clusters for the null distribution. Clusters were required to be formed by a minimum of three consecutive network thresholds. Within-group correlations with cognitive (executive function, episodic memory, processing speed, working memory, MMSE) and disease severity indices (FSRP, pack years smoked, exacerbation frequency, FEV_1_% pred., FVC % pred., PO_2_, PCO_2_ and SGRQ) were tested for the AUC_total_ analyses using partial Spearman’s Rho correlations in SPSS version 24 (IBM Corp, 2015). Correlation results were Bonferroni corrected for the number of statistical comparisons made per cognitive function or disease severity measure.

Age and sex were included as covariates of no interest in all statistical models, hereafter referred to as *confounders*. Additionally, estimated pre-morbid IQ was included in any within-group correlative model testing relationships with cognition, and total intracranial volume in all analyses using the streamline length-adjusted weighting strategy as this method did not already include a correction for head size. Pack years smoking history and anxiety and depression (HADS—total) were strongly related to group membership, therefore, it was not possible to control for these *confounders* in the statistical models. Subjects with missing cognitive or disease severity data, were excluded ‘pairwise’ from correlation analyses.

## Results

### Macrostructural brain measures

COPD patients had significantly greater normalised WMH volumes, Median (IQR) = 0.85 (1.41)% than controls = 0.40 (0.43)%, *(F*(1,49) = 5.34, *p =* 0.025). There were no group differences in total intracranial volume (COPD patients, average±SD = 1440±99, controls = 1427±81, *F*(1,24) = 2.619, *p* = 0.112. These results have been reported elsewhere [11].

### Global network analysis

The structural brain networks of all 30 patients with COPD and 23 control subjects showed ‘small-world’ topological properties (unweighted and weighted *small-worldness* >>1) across all network thresholds for both the streamline length-adjusted weighting strategy and the volume-adjusted weighting strategy (see S1 and S2 Figs). A ‘small-world’ network configuration is characterised by high clustering of local connections with a few long-range connections mediating a short path length and is thought to provide the optimal balance between modular specialisation and distributed information processing [20,31,45].

Between-group comparisons of global unweighted and weighted network metrics for the streamline length-adjusted weighting strategy made using the total AUC_total_ method are shown in Table 3. Table 4 shows the equivalent results for the comparisons made *point-by-point* along each metric curve.

**Table 3:** Group comparison of global network metrics–total area under the metric curve (AUC_total_).

**Unweighted Network** **Metrics**	**Controls**	**COPD**	***F (df***_***1***_**, *df***_***2***_***)***	***p***
Degree	154.89 (33.43)	122.76 (57.07)	8.953^2^ (1,48)	0.044^2^^b^*
Global Efficiency (x 10^−2^)	5.88 ± 0.08	5.91 ± 0.11	1.164^1^ (1,48)	1.000^1^^b^
Local Efficiency (x 10^−2^)	8.30 ± 0.31	8.15 ± 0.36	1.526^1^ (1,48)	1.000^1^^b^
Betweenness Centrality	23.00 ± 1.59	22.93 ± 5.16	0.020^1^ (1,48)	1.000^1^^b^
Small-worldness (x 10^−1^)	5.53 (1.10)	5.69 (1.36)	0.017^3^ (1,48)	0.896^3^^b^
**Weighted Network Metrics**				
Degree	36.31 ± 4.74	30.78 ± 5.89	9.584^1^ (1,48)	0.033^1^^b^*
Global Efficiency (x 10^−3^)	6.33 ± 1.27	6.48 ± 1.44	1.177^1^ (1,48)	1.000^1^^b^
Local Efficiency (x 10^−3^)	7.99 ± 1.61	7.91 ± 1.81	0.431^1^ (1,48)	1.000^1^^b^
Betweenness Centrality	45.06 (7.63)	45.53 (7.54)	0.479^2^ (1,48)	1.000^2^^b^
Small-worldness (x 10^−1^)	6.03 (1.29)	6.34 (1.60)	0.224^3^ (1,48)	0.474^3^^b^

Group comparison of global network measures using the total area under the metric curves. Age, sex and total intracranial volume were included as confounders in all analyses. Group means ± standard deviations are presented for Gaussian data, and medians (interquartile ranges) for non-Gaussian data.

^1^Gaussian and

^2^log_10_-transformed to Gaussian data were assessed using parametric ANCOVAs and non-Gaussian data by

^3^non-parametric permutation ANCOVAs (10000 permutations). *F*-statistics (*F*), degrees of freedom (*df*) and *p*-values (*p*) are displayed.

^b^Bonferroni corrected *p*-values.

*significant at *p*<0.05.

**Table 4:** Group comparison of global network metrics–‘point-by-point’ along the metric curve.

	**Peak statistics**			**Cluster**		
**Unweighted Network Metrics**	***t***_**max**_ ***(df)***	***p***_**FWE**_	***τ***	**AUC**_**MTPC**_	**AUC**_**crit**_	**MTPC**_**sig**_
Degree	3.706 (48)	0.004	17.427	15.408	5.384	Y
Global Efficiency	-2.272 (48)	0.348	0.110	-	0.002	N
Local Efficiency	2.203 (48)	0.369	0.059	-	0.003	N
Betweenness Centrality	-1.787 (48)	0.648	0.017	-	0.003	N
Small-worldness	-1.817 (48)	0.660	0.021	-	0.003	N
**Weighted Network Metrics**						
Degree	3.216 (48)	0.006	0.169	0.128	0.040	Y
Global Efficiency	-2.335 (48)	0.098	0.017	-	0.014	N
Local Efficiency	-1.779 (48)	0.420	0.021	-	0.009	N
Betweenness Centrality	-1.775 (48)	0.581	0.017	-	0.004	N
Small-worldness	-1.970 (48)	0.526	0.165	-	0.004	N

Point-by-point group comparison of global network measures. Age, sex and total intracranial volume were included as confounders in all analyses. For the maximum statistical difference (Peak), the *t*-statistic (*t*_*max*_), degrees of freedom (*df*), permutation-based family-wise error corrected *p*-value (*p*_*FWE*_) and the network threshold at which this difference occurs (*τ*) are reported under the heading ‘Peak statistics’. Additionally, the size of supra-critical clusters (AUC_MTPC_), the critical threshold for these clusters (AUC_crit_) and the significance (MTPC_sig_), Y = yes, N = no are reported under the heading ‘Cluster’.

**Fig 2:**
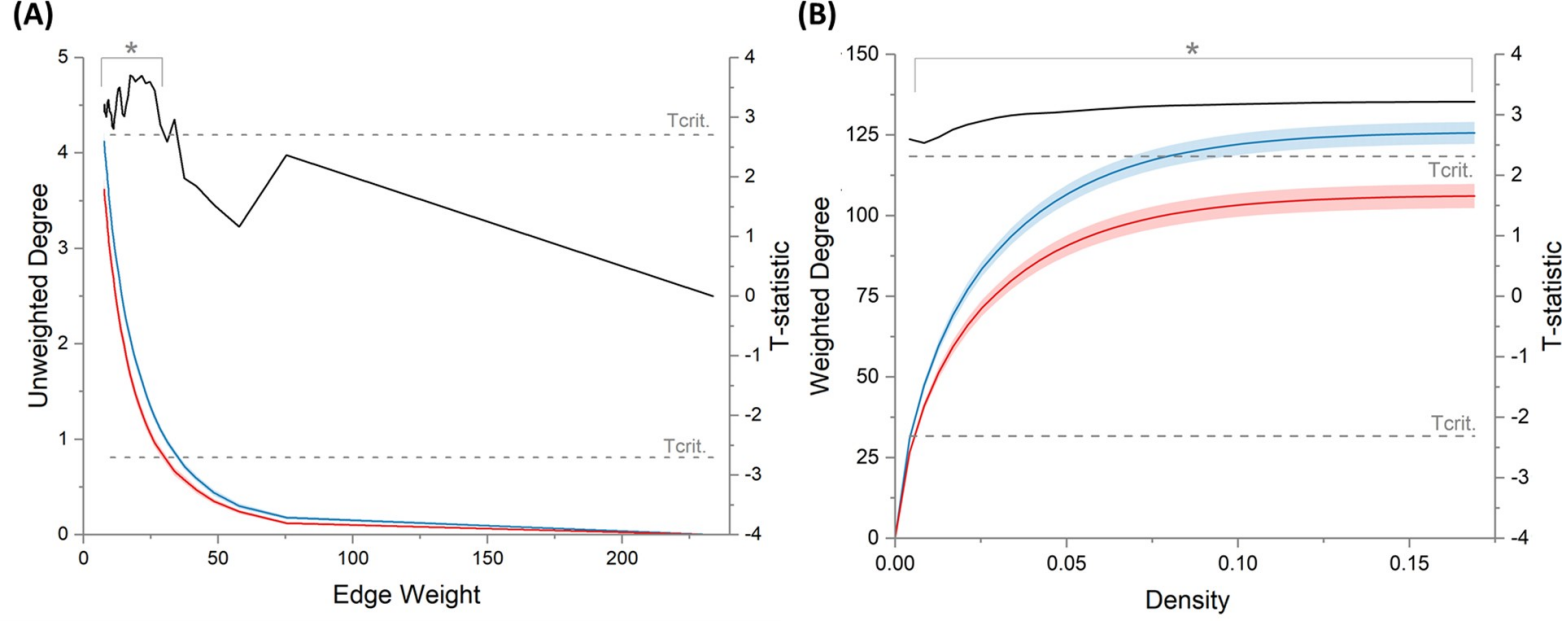
**Group comparison of unweighted degree (A) and weighted degree (B) made point-by-point along the metric curve**. Group average metric curves are plotted on the left axes. Red = COPD, Blue = Controls. Shaded error bars represent the standard error of the mean. T-statistics (black) are plotted on the right axis. Two-tailed critical thresholds (*T*_*crit*_) are indicated by dashed grey lines. *significant at *P*_*FWE*_<0.05 after MTPC correction for multiplicity.

Results for the streamline length-adjusted weighting strategy were broadly analogous between the AUC_total_ and *point-by-point* analysis approaches, with both showing that COPD patients had significantly reduced average weighted and unweighted degree compared to controls (see Tables 3 and 4). This indicates that COPD patients’ brain networks contained fewer edges and that edges were generally *weaker* i.e. composed of fewer streamlines. However, the *point-by-point* approach also showed that, whilst patients with COPD had significantly reduced average weighted degree across all network thresholds, differences in average unweighted degree were confined to low edge weighting thresholds. These findings suggest that the brain networks of COPD patients contain fewer *weak* network edges but a similar number of *strong* edges to those present in controls (Fig 2 and Table 4). Use of the volume-adjusted weighting strategy removed the significance of the group differences in the AUC_total_ of average weighted and unweighted degree, although a trend remained, *F*(1,49) = 7.722, *p =* 0.080 and *F*(1,49) = 8.016, *p* = 0.067, respectively (see S1 Table), however, both remained significant using the *point-by-point* approach (*t*_max_(49) = 3.653, *p* = 0.005 and *t*_max_(49) = 2.822, *p =* 0.018, respectively, see S2 Table).

There were no other significant group differences for the remaining weighted or unweighted global network metrics, including: global efficiency, local efficiency, betweenness centrality or *small-worldness*, regardless of the edge weighting strategy used (Tables 3 and 4 and S1 and S2 and S1 and S2 Figs).

### Nodal network analysis

**Fig 3:**
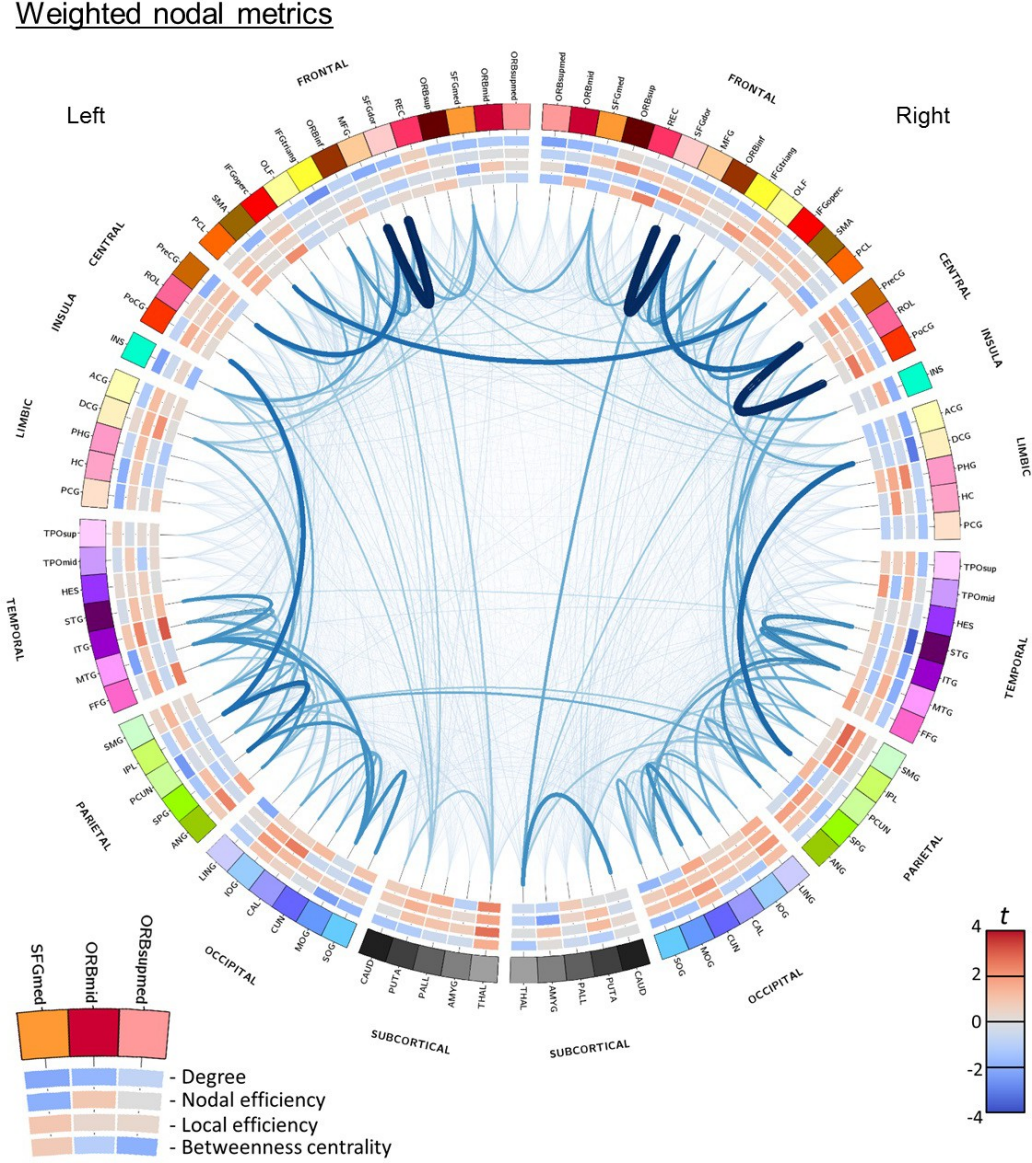
Circular representation of network connections in all subjects and between-group differences in weighted nodal network metrics. Network nodes are arranged around the outermost circle and assigned a unique colour. Nodes are split by hemisphere (right hemisphere on the right) and grouped within the macroscopic subdivisions defined in [32] (Frontal, Central, Insula, Limbic, Temporal, Parietal, Occipital, Subcortical). Within these subdivisions nodes are arranged by structural laterality. S3 Table summarises the node name abbreviations. The inner four circles show red-blue *t*-statistic heatmaps for the sub-significant between-group AUC_total_ trends in nodal weighted metrics for the contrast COPD>Controls. Connections represent the edges present in any subject. The thickness and darkness of connections indicates the average edge weight for the streamline length-adjusted weighting strategy.

The spatial pattern of trends in the weighted nodal metrics for the streamline length-adjusted weighting strategy using the AUC_total_ method can be viewed in Fig 3. An equivalent figure for the unweighted metric results and for the volume-adjusted weighting strategy can be found in the S3 and S4 Figs. There was a general trend for patients with COPD to have numerically lower weighted and unweighted nodal degree (71/90 and 70/90 of nodes, respectively), and numerically higher weighted nodal efficiency (72/90 nodes). However, the only significant difference occurred for the nodal unweighted degree in the right hemispheric superior temporal gyrus, with COPD patients having lower unweighted degree than controls (*t*(48) = -3.961, *p* = 0.016). This result did not survive correction for end-node volume. No nodal group differences were found using the MTPC method.

### Correlations with cognitive function and disease severity

There were no significant partial spearman’s rho correlations for weighted or unweighted global network measures and disease severity or cognitive function for either subject group, regardless of the edge weighting strategy used (Tables 5 and S4–S6).

**Table 5:** Within-group correlations between global weighted network metrics and cognitive and disease severity measures.

Weighted Global Network Metrics										
	Degree		Global Efficiency		Local Efficiency		Betweenness Centrality		Small-worldness	
**Controls (N = 23)**	*rho (df)*	*p*	*rho (df)*	*p*	*rho (df)*	*p*	*rho (df)*	*p*	*rho (df)*	*p*
Executive Function	0.561 (18)	0.100^b^	-0.007 (18)	1.000^b^	-0.078 (18)	1.000^b^	0.265 (18)	1.000^b^	0.176 (18)	1.000^b^
Episodic Memory	0.003 (18)	1.000^b^	-0.085 (18)	1.000^b^	0.079 (18)	1.000^b^	-0.367 (18)	1.000^b^	0.510 (18)	0.220^b^
Processing Speed	0.337 (18)	1.000^b^	-0.049 (18)	1.000^b^	-0.080 (18)	1.000^b^	-0.133 (18)	1.000^b^	0.410 (18)	0.720^b^
Working Memory	0.452 (18)	0.460^b^	-0.071 (18)	1.000^b^	-0.108 (18)	1.000^b^	0.019 (18)	1.000^b^	0.156 (18)	1.000^b^
MMSE	0.054 (18)	1.000^b^	-0.283 (18)	1.000^b^	-0.078 (18)	1.000^b^	-0.012 (18)	1.000^b^	0.194 (18)	1.000^b^
**COPD (N = 30)**										
Executive Function	-0.238 (25)	1.000^b^	0.225 (25)	1.000^b^	0.105 (25)	1.000^b^	-0.170 (25)	1.000^b^	-0.245 (25)	1.000^b^
Episodic Memory	-0.006 (25)	1.000^b^	-0.330 (25)	0.930^b^	-0.390 (25)	0.440^b^	-0.366 (25)	0.600^b^	-0.110 (25)	1.000^b^
Processing Speed	-0.138 (25)	1.000^b^	-0.101 (25)	1.000^b^	-0.112 (25)	1.000^b^	-0.149 (25)	1.000^b^	-0.107 (25)	1.000^b^
Working Memory	0.025 (25)	1.000^b^	0.065 (25)	1.000^b^	0.005 (25)	1.000^b^	-0.237 (25)	1.000^b^	-0.237 (25)	1.000^b^
MMSE	0.049 (25)	1.000^b^	0.108 (25)	1.000^b^	0.233 (25)	1.000^b^	0.015 (25)	1.000^b^	0.291 (25)	1.000^b^
FSRP	-0.081 (26)	1.000^b^	-0.018 (26)	1.000^b^	-0.043 (26)	1.000^b^	0.157 (26)	1.000^b^	0.098 (26)	1.000^b^
Pack Years	-0.067 (26)	1.000^b^	0.098 (26)	1.000^b^	0.186 (26)	1.000^b^	-0.047 (26)	1.000^b^	-0.095 (26)	1.000^b^
Exacerbation Frequency	0.033 (26)	1.000^b^	-0.278 (26)	1.000^b^	-0.224 (26)	1.000^b^	-0.110 (26)	1.000^b^	0.318 (26)	1.000^b^
FEV_1_ (% pred.)	0.139 (26)	1.000^b^	-0.446 (26)	0.200^b^	-0.403 (26)	0.370^b^	-0.045 (26)	1.000^b^	0.093 (26)	1.000^b^
FVC (% pred.)	0.144 (26)	1.000^b^	-0.311 (26)	1.000^b^	-0.199 (26)	1.000^b^	-0.275 (26)	1.000^b^	0.368 (26)	0.590^b^
PO_2_	-0.135 (26)	1.000^b^	-0.074 (26)	1.000^b^	-0.045 (26)	1.000^b^	-0.075 (26)	1.000^b^	-0.012 (26)	1.000^b^
PCO_2_	0.092 (26)	1.000^b^	0.353 (26)	0.650^b^	0.250 (26)	1.000^b^	0.146 (26)	1.000^b^	-0.032 (26)	1.000^b^
SGRQ	0.068 (26)	1.000^b^	0.223 (26)	1.000^b^	0.239 (26)	1.000^b^	0.082 (26)	1.000^b^	-0.010 (26)	1.000^b^

Age, sex and total intracranial volume were entered as confounders in all analyses. Additionally, estimated pre-morbid IQ was included in correlations involving cognitive function. Spearman’s correlation coefficients (*rho*), and *p*-values (*p*) are displayed.

^b^Bonferroni corrected *p*-values.

## Discussion

This study used deterministic tractography and structural network analysis to investigate cross-sectionally whether there are any differences in the pattern of white matter connectivity between people with COPD and control subjects. Both subject groups’ networks had the expected ‘small-world’ topology, with high clustering of local connections and short path length. Globally, COPD patients’ networks were found to have reduced white matter connectivity both in terms of the number of network edges and the *strength* of these edges (the adjusted number of constituent tractography streamlines). Analysis on a *point-by-point* basis indicated that the strength of network edges was reduced across all levels of network density, whereas the difference in the number of edges only occurred at low edge weighting thresholds. This suggests that whilst both *strong* and *weak* network edges were impaired, only *weak* edges were disconnected entirely. In contrast, there were no significant group differences in the topological organisation of the networks (i.e. no difference in network integration, segregation, nodal influence or *small-worldness*), this suggests that there is sufficient redundancy to accommodate a reduction in connectivity without compromising the organisational efficiency of the overall network structure. These results appear to have been driven by subtle (and largely sub-significant) group differences across the majority of network nodes. Adjusting for differences in end-node volume (volume-adjusted weighting strategy) removed the significance of group differences in average unweighted and weighted degree when measured as the total area under the metric curves, however, they remained significant when using the *point-by-point* approach. This suggests that even if local cerebral atrophy contributed to these group differences it is unlikely to be fully responsible for them. There were no other notable differences between the results from the two weighting strategies. No significant relationships were found between global network metrics and measures of cognitive function or disease severity, so it cannot be determined whether or not loss of connectivity was responsible for cognitive dysfunction in this cohort.

To our knowledge this is the first study to use structural network analysis to examine white matter connectivity in people with COPD. However, the finding of reductions in white matter connectivity in COPD compared to controls are consistent with reports of widespread tract-based increases in diffusivity and decreases in anisotropy, found both in this and other COPD cohorts [10,12,13], suggestive of a deterioration in the white matter in COPD. Such diffusion abnormalities are consistent with those found in other chronic diseases with a high prevalence of mild cognitive impairment, including diabetes, hypertension and chronic kidney disease e.g. [16,46–50]. A previous study of the present cohort found increases in functional connectivity, encompassing all resting-state networks except the visual network [10]. It is plausible that this increase in functional connectivity reflects an over-recruitment of the surviving network structure in order to compensate for the loss of white matter connectivity.

Since no relationships were found with clinical disease severity measures in this study, it is difficult to draw any mechanistic conclusions about the pathophysiological causes of this network disruption. However, a number of conventional anatomical MR imaging markers of cerebral SVD, including small subcortical infarcts, WMHs, cerebral microbleeds and brain atrophy (including generalised atrophy, ventriculomegaly, hippocampal atrophy and focal atrophy) [51] have been identified in COPD [8–10,13,52–54] or found to be associated with reduced lung function [55–58]. Furthermore, cardiovascular risk factors which are thought to predispose development of arteriolosclerosis (otherwise known as age-related cardiovascular risk-factor-related SVD) are also commonly found in COPD—most notably elderly age, diabetes, hypertension and smoking [51,59,60]. Additionally, COPD itself is an independent risk factor for cardiovascular disease [61]. Consequently, it has been hypothesised that SVD is responsible for the neuropathology and cognitive impairment found in COPD [8,9,11,62].

Previous network studies of SVD have reported reductions in the density and *strength* of network edges [21,63] consistent with those found in the present study. However, they also report more profound alterations to network topology not present in this study, such as reductions in network integration and segregation [21,62,63]. These topological changes have been found to mediate the relationship between DTI measures of white matter deterioration and cognitive impairment (executive function and processing speed) [21]. Therefore, the lack of substantial disruption in network topology in the present cohort may account for the mildness of their cognitive impairment (only one patient met the MMSE criteria for severe cognitive impairment) as their brain networks are able to accommodate a loss of connections without compromising the overall efficiency of the network. Nevertheless this loss of connections is likely to increase the vulnerability of the networks to future damage and increase the risk of further cognitive decline. The potential utility of brain MR features as early prognostic markers of cognitive decline and dementia onset have been demonstrated in other diseases [64–67]. For instance, a prospective study showed that longitudinal change in MR measures, including increase in WMHs and worsening of white matter tissue microstructure (measured using DTI) were predictive of conversion to dementia in SVD, despite there being no detectable change on neuropsychological testing [66]. Furthermore, longitudinal decline in white matter connectivity was found to mediate many of the relationships between progression of conventional brain MR and DTI markers of SVD and conversion to dementia [67]. Future investigation of longitudinal change in white matter connectivity in COPD may help predict which patients are at greater risk of developing cognitive impairment and dementia, enabling personalised treatment and support.

The lack of direct relationships between cognitive and network measures in the present study is perhaps surprising, given that a number of other studies have reported relationships between disruption of large-scale structural brain networks and reduced cognitive function e.g. [21,63, 68,69]. This may reflect the relatively small cohort size in this study and/or the mild severity of the COPD (moderate-severe airflow obstruction without significant hypoxaemia or hypercapnia) in this study. Brain reserve capacity [70] and cognitive reserve [71] have been proposed to explain similar disparities in brain pathology and functional outcome [71,72]. These related concepts suggest that an individual’s trajectory of cognitive decline is moderated by factors, such as the amount of physical substrate available (e.g. brain size, number of neurons) and how effectively they can utilise their brain networks (e.g. cognitive efficiency and flexibility) [72]; this may affect their resilience to accumulating pathology. In this study estimated pre-morbid IQ and total intracranial volume were included as covariates in statistical analyses as surrogates for cognitive reserve [73] and brain reserve e.g. [74]. However, it is possible that residual effects remained. A number of disease-related factors and co-morbidities including anxiety and depression [75,76], disturbed sleep [76,77] and reduced physical activity [78,79] may also be contributing to functional impairment without commensurate effects on white matter structure. Further research is required to elucidate the relationship between disease-related factors, changes in white matter connectivity and cognitive impairment in COPD.

## Limitations

The main limitation of this study was that it was not possible to adequately control for smoking history, hypertension or anxiety and depression in the between-group analysis due to the strong dependence of number of pack years smoked and total HADS score on group-membership, and the lack of available blood pressure data. Consequently, it was not possible to exclude these disease factors as being responsible for the network disruption. This is particularly significant as previous studies have reported DTI and/or structural and functional network abnormalities to occur with these conditions e.g. [16,80–82]. No formal power calculation was performed prior to data acquisition. The sample size is relatively small, although comparable in size to other studies that have reported group differences in MR imaging measures in COPD [10,13,53,83], limiting the generalisability of these findings to other COPD cohorts. This study used composite measures of cognition to test for correlations with network measures. This is a common approach used e.g. [21,63,84], however, it is possible that individual neuropsychological sub-tests would have been more sensitive to network disruption.

Diffusion MR remains the only non-invasive in vivo method for investigating white matter connectivity. With deterministic tractography the uncertainty of the principal direction of diffusion in areas of low FA (e.g. in WMHs) or complex fibre anatomy (e.g. the crossing, bending, and kissing fibres prevalent in the optic radiation, callosal fibres, pyramidic tracts [85]) lead to errors which accumulate along the length of the tractography streamline. The present study aimed to reduce these effects by only seeding the deterministic tractography algorithm within areas with a well-defined principal diffusion direction (FA≥0.2) [21] and by using super-resolution seeding to reduce partial-volume effects [36]. Application of probabilistic tractography (e.g. [86]) and/or a correction for cerebrospinal fluid contamination may improve tracking through areas of low FA [87,88] such as the WMHs present in this cohort and areas of partial-voluming with cerebrospinal fluid. However, probabilistic tractography is more computationally demanding and has an elevated risk of producing false-positive connections [89]. Constrained spherical deconvolution [90,91] based tractography could also be used to overcome some of these effects, but has limited applicability to the present data which used relatively few diffusion directions at b = 1000s mm^-2^ [92].

## Conclusions

This study has provided a cross-sectional analysis of differences in white matter connectivity between COPD patients with mild-moderate airflow obstruction and age and sex-matched controls. Compared to controls, COPD patients had under-connected structural networks comprising fewer and *weaker* network connections, but with their topological organisation conserved. It was not possible to remove the confounding effects of smoking history and hypertension, so it could not be determined whether this was COPD-related effect *per se*, or whether it was the result of COPD patients having greater cardiovascular risk.

## Supporting information

S1 Table Group comparison of global network metrics for the volume-adjusted weighting strategy–total area under the metric curve. Group comparison of global network measures using the total area under the metric curves. Age and sex were included as confounders in all analyses. Group means ± standard deviations are presented for Gaussian data, and medians (interquartile ranges) for non-Gaussian data. ^1^Gaussian and ^2^log_10_-transformed to Gaussian data were assessed using parametric ANCOVAs and non-Gaussian data by ^3^non-parametric permutation ANCOVAs (10000 permutations). *F*-statistics, degrees of freedom (*df*_*1*_, *df*_*2*_) and *p*-values are displayed.
(DOCX)

S2 Table Group comparison of global network metrics for the volume-adjusted weighting strategy–‘point-by-point’ along the metric curve. Point-by-point group comparison of global network measures. Age and sex were included as confounders in all analyses. For the maximum statistical difference (Peak), the *t*-statistic (*t*_*max*_), degrees of freedom (*df*), permutation-based family-wise error corrected *p-*value (*p*_*FWE*_) and the network threshold at which this difference occurs (*τ*) are reported under the heading ‘Peak statistics’. Additionally, the size of supra-critical clusters (AUC_MTPC_), the critical threshold for these clusters (AUC_crit_) and their significance (MTPC_sig_), Y = yes, N = no are reported under the heading ‘Cluster’.
(DOCX)

S3 Table List of network nodes and abbreviations for the circular network diagrams. (DOCX)

S4 Table Within-group correlations between global unweighted network metrics and cognitive and disease severity measures for the streamline length-adjusted weighting strategy. Age, sex and total intracranial volume were entered as confounder in all analyses. Additionally, estimated pre-morbid IQ was included in correlations involving cognitive function. Spearman’s correlation coefficients (*rho*), degrees of freedom (*df*) and *p*-values (*p*) are displayed. ^b^Bonferroni corrected *p*-values. =
(DOCX)

S5 Table Within-group correlations between global weighted network metrics and cognitive and disease severity measures for the volume-adjusted weighting strategy. Age and sex were entered as confounders in all analyses. Additionally, estimated pre-morbid IQ was included in correlations involving cognitive function. Spearman’s correlation coefficients (*rho*), degrees of freedom (*df*) and *p*-values (*p*) are displayed. ^b^Bonferroni corrected *p*-values.
(DOCX)

S6 Table Within-group correlations between global unweighted network metrics and cognitive and disease severity measures for the volume-adjusted weighting strategy. Age and sex were entered as confounders in all analyses. Additionally, estimated pre-morbid IQ was included in correlations involving cognitive function. Spearman’s correlation coefficients (*rho*), degrees of freedom (*df*) and p-values (*p*) are displayed. ^b^Bonferroni corrected *p*-values.
(DOCX)

S1 Fig Group comparison of unweighted (left column) and weighted (right column) global network metrics made point-by-point along the metric curve for the streamline length-adjusted weighting strategy. Group average metric curves for unweighted and weighted global network metrics are plotted on the left axes. Red = COPD patients, Blue = Controls. Shaded error bars represent the standard error of the mean. *T*-statistics (black) are plotted on the right axis. Two-tailed critical thresholds (*T*_*crit*_) are indicated by dashed grey lines.
(PDF)

S2 Fig Group comparison of unweighted (left column) and weighted (right column) global network metrics made point-by-point along the metric curve for the volume-adjusted weighting strategy. Group average metric curves for unweighted and weighted global network metrics are plotted on the left axes. Red = COPD patients, Blue = Controls. Shaded error bars represent the standard error of the mean. *T*-statistics (black) are plotted on the right axis. Two-tailed critical thresholds (*T*_*crit*_) are indicated by dashed grey lines. *significant at *P*_*FWE*_<0.05 after MTPC correction for multiplicity.
(PDF)

S3 Fig Circular representation of network connections in all subjects and between-group differences in unweighted nodal network metrics for the streamline length-adjusted weighting strategy. Network nodes are arranged around the outermost circle and assigned a unique colour. Nodes are split by hemisphere (right hemisphere on the right) and grouped within the macroscopic subdivisions defined in [32] (Frontal, Central, Insula, Limbic, Temporal, Parietal, Occipital, Subcortical). Within these subdivisions nodes are arranged by structural laterality. S3 Table summarises the node name abbreviations. The inner four circles show red-blue t-statistic heatmaps for between-group AUC_total_ differences in nodal unweighted metrics for the contrast COPD patients>controls. Connections represent the edges present in any subject. The thickness and darkness of connections indicates the average edge weight for the streamline length-adjusted weighting strategy. Significant results are outlined in black.
(TIF)

S4 Fig Circular representation of network connections in all subjects and between-group differences in nodal network metrics for the volume-adjusted weighting strategy. Network nodes are arranged around the outermost circle and assigned a unique colour. Nodes are split by hemisphere (right hemisphere on the right) and grouped within the macroscopic subdivisions defined in [32] (Frontal, Central, Insula, Limbic, Temporal, Parietal, Occipital, Subcortical). Within these subdivisions nodes are arranged by structural laterality. S3 Table summarises the node name abbreviations. The inner four circles show red-blue t-statistic heatmaps for the sub-significant between-group AUC_total_ trends in nodal unweighted metrics for the contrast COPD patients>controls. Connections represent the edges present in any subject. The thickness and darkness of connections indicates the average edge weight for the volume-adjusted weighting strategy.
(TIF)

S1 File Study data. (XLSX)

## Abbreviations

AUC: Area under the Curve
COPD: Chronic Obstructive Pulmonary Disease
DTI: Diffusion Tensor Images
DWI: Diffusion Weighted Images
FA: Fractional Anisotropy
FEV_1_: Forced Expiratory Volume in one second
FSRP: Modified Framingham Stroke Risk Profile
FVC: Forced Vital Capacity
FEW: Family-Wise Error
HADS: Hospital Anxiety and Depression Scale
MNI: Montreal Neurological Institute
MMSE: Mini Mental State Examination
MR: Magnetic Resonance
MTPC: Multi-Threshold Permutation Correction
PCO_2_: Partial Pressure of Carbon Dioxide
PO_2_: Partial Pressure of Oxygen
SGRQ: St George’s Respiratory Questionnaire
SVD: Cerebral Small Vessel Disease
τ: Network Threshold
t_max_: Peak T-statistic
WMH(s): White Matter Hyperintensities of Presumed Vascular Origin
