# Supplementary material for: Disruption of white matter connectivity in chronic obstructive pulmonary disease
Source: PLoS One. 2019 Oct 3;14(10):e0223297. doi: 10.1371/journal.pone.0223297 (PMC6776415; doi:10.1371/journal.pone.0223297)
Supplement: S1 Table — Group comparison of global network measures using the total area under the metric curves. Age and sex were included as confounders in all analyses. Group means ± standard deviations are presented for Gaussian data, and medians (interquartile ranges) for non-Gaussian data. 1Gaussian and 2log10-transformed to Gaussian data were assessed using parametric ANCOVAs and non-Gaussian data by 3non-parametric permutation ANCOVAs (10000 permutations). F-statistics, degrees of freedom (df1, df2) and p-values are displayed. (DOCX) [file pone.0223297.s001.docx]

**S1 Table: Group comparison of global network metrics for the volume-adjusted weighting strategy – total area under the metric curve**

| **Unweighted Network Metrics** | **Normal Controls** | **COPD Patients** | ***F (df_1_,df_2_)*** | ***p*** |
| --- | --- | --- | --- | --- |
| Degree | 1.51 ± 0.07 | 1.43 ± 0.10 | 8.016 (1,49) | 0.067^1b^ |
| Global Efficiency (x 10^-2^) | 7.24 ± 0.01 | 7.29 ± 0.01 | 2.790 (1,49) | 1.000^1b^ |
| Local Efficiency (x 10^-2^) | 9.58 ± 0.41 | 9.42 ± 0.42 | 2.058 (1,49) | 1.000^1b^ |
| Betweenness Centrality | 25.69 ± 2.01 | 25.57 ± 1.43 | 0.089 (1,49) | 1.000^1b^ |
| Small-worldness (x 10^-1^) | 5.01 (1.12) | 4.45 (0.94) | 0.599 (1,49) | 1.000^3b^ |
| Weighted Network Metrics |  |  |  |  |
| Degree (x 10^-2^) | 9.21 ± 1.17 | 8.08 ± 1.52 | 7.722 (1,49) | 0.080^1b^ |
| Global Efficiency (x 10^-3^) | 9.77 ± 3.20 | 9.87 ± 2.94 | 0.014 (1,49) | 1.000^1b^ |
| Local Efficiency (x 10^-2^) | 1.01 ± 0.35 | 0.96 ± 0.32 | 0.623 (1,49) | 1.000^1b^ |
| Betweenness Centrality | 47.31 ± 60.07 | 47.78 ± 4.51 | 0.053 (1,49) | 1.000^1b^ |
| Small-worldness (x 10^-1^) | 5.26 ± 0.90 | 5.14 ± 1.09 | 0.603 (1,49) | 1.000^1b^ |

Group comparison of global network measures using the total area under the metric curves. Age and sex were included as confounders in all analyses. Group means ± standard deviations are presented for Gaussian data, and medians (interquartile ranges) for non-Gaussian data. ^1^Gaussian and ^2^log_10_-transformed to Gaussian data were assessed using parametric ANCOVAs and non-Gaussian data by ^3^non-parametric permutation ANCOVAs (10000 permutations). *F*-statistics, degrees of freedom (*df_1_, df_2_*) and *p*-values are displayed.
