# Supplementary material for: Disruption of white matter connectivity in chronic obstructive pulmonary disease
Source: PLoS One. 2019 Oct 3;14(10):e0223297. doi: 10.1371/journal.pone.0223297 (PMC6776415; doi:10.1371/journal.pone.0223297)
Supplement: S3 Table — (DOCX) [file pone.0223297.s003.docx]

**S3 Table. List of network nodes and abbreviations for the circular network diagrams**

| **Macroscopic anatomical region** | **Node name** | **Abbreviation** |
| --- | --- | --- |
| Frontal lobe | Superior medial orbital gyrus | ORBsupmed |
|  | Middle orbital gyrus | ORBmid |
|  | Medial superior frontal gyrus | SFGmed |
|  | Superior orbital gyrus | ORBsup |
|  | Gyrus rectus | REC |
|  | Dorsal superior frontal gyrus | SFGdor |
|  | Middle frontal gyrus | MFG |
|  | Inferior frontal gyrus, *pars orbitalis* | ORBinf |
|  | Inferior frontal gyrus, *pars triangularis* | IFGtriang |
|  | Olfactory cortex | OLF |
|  | Inferior frontal gyrus, *pars opercularis* | IFGoperc |
|  | Supplementary motor area | SMA |
|  | Paracentral lobule | PCL |
| Central region | Precentral gyrus | PreCG |
|  | Rolandic operculum | ROL |
|  | Postcentral gyrus | PoCG |
| Insula | Insula | INS |
| Limbic lobe | Anterior cingulate gyrus | ACG |
|  | Dorsal cingulate gyrus | DCG |
|  | Parahippocampal gyrus | PHG |
|  | Hippocampus | HC |
|  | Posterior cingulate gyrus | PCG |
| Temporal lobe | Superior temporal pole | TPOsup |
|  | Middle temporal pole | TPOmid |
|  | Heschl gyrus | HES |
|  | Superior temporal gyrus | STG |
|  | Inferior temporal gyrus | ITG |
|  | Middle temporal gyrus | MTG |
|  | Fusiform gyrus | FFG |
| Parietal lobe | Supramarginal gyrus | SMG |
|  | Inferior parietal lobe | IPL |
|  | Precuneus | PCUN |
|  | Superior parietal gyrus | SPG |
|  | Angular gyrus | ANG |
| Occipital lobe | Lingual gyrus | LING |
|  | Inferior occipital gyrus | IOG |
|  | Peri-calcarine cortex | CAL |
|  | Cuneus | CUN |
|  | Middle occipital gyrus | MOG |
|  | Superior occipital gyrus | SOG |
| Subcortical grey nuclei | Caudate nucleus | CAUD |
|  | Putamen | PUTA |
|  | Pallidum | PALL |
|  | Amygdala | AMYG |
|  | Thalamus | THAL |
