# Supplementary material for: Disruption of white matter connectivity in chronic obstructive pulmonary disease
Source: PLoS One. 2019 Oct 3;14(10):e0223297. doi: 10.1371/journal.pone.0223297 (PMC6776415; doi:10.1371/journal.pone.0223297)
Supplement: S4 Table — Age, sex and total intracranial volume were entered as confounder in all analyses. Additionally, estimated pre-morbid IQ was included in correlations involving cognitive function. Spearman’s correlation coefficients (rho), degrees of freedom (df) and p-values (p) are displayed. bBonferroni corrected p-values. = (DOCX) [file pone.0223297.s004.docx]

**S4 Table. Within-group correlations between global unweighted network metrics and cognitive and disease severity measures for the streamline length-adjusted weighting strategy**

| **Unweighted Global Network Metrics** | | | | | | | | | | |
| --- | --- | --- | --- | --- | --- | --- | --- | --- | --- | --- |
|  | **Degree** | | **Global Efficiency** | | **Local Efficiency** | | **Betweenness Centrality** | | **Small-worldness** | |
| **Controls (N=23)** | *rho (df)* | *p* | *rho (df)* | *p* | *rho (df)* | *p* | *rho (df)* | *p* | *rho (df)* | *p* |
| Executive Function | 0.473 (18) | 0.350^b^ | -0.139 (18) | 1.000^b^ | 0.123 (18) | 1.000^b^ | 0.409 (18) | 0.730^b^ | 0.206 (18) | 1.000^b^ |
| Episodic Memory | 0.051 (18) | 1.000^b^ | 0.141 (18) | 1.000^b^ | -0.010 (18) | 1.000^b^ | -0.077 (18) | 1.000^b^ | 0.501 (18) | 0.240^b^ |
| Processing Speed | 0.387 (18) | 0.920^b^ | 0.045 (18) | 1.000^b^ | 0.096 (18) | 1.000^b^ | 0.116 (18) | 1.000^b^ | 0.441 (18) | 0.510^b^ |
| Working Memory | 0.340 (18) | 1.000^b^ | -0.026 (18) | 1.000^b^ | -0.217 (18) | 1.000^b^ | 0.081 (18) | 1.000^b^ | 0.281 (18) | 1.000^b^ |
| MMSE | -0.091 (18) | 1.000^b^ | -0.250 (18) | 1.000^b^ | -0.066 (18) | 1.000^b^ | 0.067 (18) | 1.000^b^ | 0.202 (18) | 1.000^b^ |
| **COPD Patients (N=30)** | | | | | | | | | | |
| Executive Function | -0.252 (25) | 1.000^b^ | 0.289 (25) | 1.000^b^ | -0.296 (25) | 1.000^b^ | -0.160 (25) | 1.000^b^ | -0.208 (25) | 1.000^b^ |
| Episodic Memory | -0.100 (25) | 1.000^b^ | 0.256 (25) | 1.000^b^ | -0.367 (25) | 0.600^b^ | -0.470 (25) | 0.130^b^ | -0.074 (25) | 1.000^b^ |
| Processing Speed | 0.067 (25) | 1.000^b^ | 0.092 (25) | 1.000^b^ | -0.371 (25) | 0.570^b^ | -0.306 (25) | 1.000^b^ | -0.038 (25) | 1.000^b^ |
| Working Memory | -0.063 (25) | 1.000^b^ | 0.178 (25) | 1.000^b^ | -0.339 (25) | 0.840^b^ | -0.267 (25) | 1.000^b^ | -0.195 (25) | 1.000^b^ |
| MMSE | 0.035 (25) | 1.000^b^ | 0.061 (25) | 1.000^b^ | 0.109 (25) | 1.000^b^ | 0.141 (25) | 1.000^b^ | 0.324 (25) | 0.990^b^ |
| FRSP | -0.021 (26) | 1.000^b^ | 0.078 (26) | 1.000^b^ | 0.380 (26) | 0.460^b^ | 0.104 (26) | 1.000^b^ | -0.212 (26) | 1.000^b^ |
| Pack Years | -0.180 (26) | 1.000^b^ | 0.221 (26) | 1.000^b^ | -0.086 (26) | 1.000^b^ | 0.050 (26) | 1.000^b^ | -0.068 (26) | 0.680^b^ |
| Exacerbation Frequency | 0.031 (26) | 1.000^b^ | 0.060 (26) | 1.000^b^ | 0.036 (26) | 1.000^b^ | -0.011 (26) | 1.000^b^ | 0.350 (26) | 1.000^b^ |
| FEV_1_ (% pred.) | 0.139 (26) | 1.000^b^ | -0.305 (26) | 1.000^b^ | 0.202 (26) | 1.000^b^ | -0.015 (26) | 1.000^b^ | 0.039 (26) | 1.000^b^ |
| FVC (% pred.) | 0.068 (26) | 1.000^b^ | -0.213 (26) | 1.000^b^ | 0.163 (26) | 1.000^b^ | -0.044 (26) | 1.000^b^ | 0.398 (26) | 0.430^b^ |
| PO_2_ | -0.185 (26) | 1.000^b^ | -0.014 (26) | 1.000^b^ | -0.208 (26) | 1.000^b^ | -0.029 (26) | 1.000^b^ | -0.106 (26) | 1.000^b^ |
| PCO_2_ | 0.085 (26) | 1.000^b^ | 0.298 (26) | 1.000^b^ | 0.108 (26) | 1.000^b^ | 0.159 (26) | 1.000^b^ | -0.006 (26) | 1.000^b^ |
| SGRQ | 0.083 (26) | 1.000^b^ | 0.041 (26) | 1.000^b^ | 0.061 (26) | 1.000^b^ | 0.158 (26) | 1.000^b^ | -0.006 (26) | 1.000^b^ |

Age, sex and total intracranial volume were entered as confounder in all analyses. Additionally, estimated pre-morbid IQ was included in correlations involving cognitive function. Spearman’s correlation coefficients (*rho*), degrees of freedom (*df*) and *p*-values (*p*) are displayed. ^b^Bonferroni corrected *p*-values.
