# Supplementary material for: Disruption of white matter connectivity in chronic obstructive pulmonary disease
Source: PLoS One. 2019 Oct 3;14(10):e0223297. doi: 10.1371/journal.pone.0223297 (PMC6776415; doi:10.1371/journal.pone.0223297)
Supplement: S5 Table — Age and sex were entered as confounders in all analyses. Additionally, estimated pre-morbid IQ was included in correlations involving cognitive function. Spearman’s correlation coefficients (rho), degrees of freedom (df) and p-values (p) are displayed. bBonferroni corrected p-values. (DOCX) [file pone.0223297.s005.docx]

**S5 Table. Within-group correlations between global weighted network metrics and cognitive and disease severity measures for the volume-adjusted weighting strategy**

| **Weighted Global Network Metrics** | | | | | | | | | | |
| --- | --- | --- | --- | --- | --- | --- | --- | --- | --- | --- |
|  | **Degree** | | **Global Efficiency** | | **Local Efficiency** | | **Betweenness Centrality** | | **Small-worldness** | |
| **Controls (N=23)** | *rho (df)* | *p* | *rho (df)* | *p* | *rho (df)* | *p* | *rho (df)* | *p* | *rho (df)* | *p* |
| Executive Function | 0.535 (19) | 0.152^b^ | -0.384 (19) | 0.948 | -0.363 (19) | 1.000^b^ | 0.442 (19) | 0.511^b^ | 0.091 (19) | 1.000^b^ |
| Episodic Memory | 0.251 (19) | 1.000^b^ | 0.181 (19) | 1.000^b^ | 0.200 (19) | 1.000^b^ | -0.013 (19) | 1.000^b^ | 0.224 (19) | 1.000^b^ |
| Processing Speed | 0.486 (19) | 0.297^b^ | 0.000 (19) | 1.000^b^ | -0.058 (19) | 1.000^b^ | 0.163 (19) | 1.000^b^ | 0.067 (19) | 1.000^b^ |
| Working Memory | 0.495 (19) | 0.264^b^ | -0.157 (19) | 1.000^b^ | -0.130 (19) | 1.000^b^ | -0.192 (19) | 1.000^b^ | -0.362 (19) | 1.000^b^ |
| MMSE | -0.001 (19) | 1.000^b^ | 0.125 (19) | 1.000^b^ | 0.179 (19) | 1.000^b^ | -0.020 (19) | 1.000^b^ | 0.211 (19) | 1.000^b^ |
| **COPD Patients (N=30)** | | | | | | | | | | |
| Executive Function | -0.033 (26) | 1.000^b^ | -0.347 (26) | 0.764^b^ | -0.382 (26) | 0.493^b^ | -0.100 (26) | 1.000^b^ | -0.124 (26) | 1.000^b^ |
| Episodic Memory | 0.309 (26) | 1.000^b^ | -0.503 (26) | 0.075^b^ | -0.445 (26) | 0.149^b^ | -0.343 (26) | 0.803^b^ | -0.368 (26) | 0.591^b^ |
| Processing Speed | 0.370 (26) | 0.576^b^ | -0.222 (26) | 1.000^b^ | -0.238 (26) | 1.000^b^ | -0.222 (26) | 1.000^b^ | -0.287 (26) | 1.000^b^ |
| Working Memory | 0.287 (26) | 1.000^b^ | -0.237 (26) | 1.000^b^ | -0.242 (26) | 1.000^b^ | -0.223 (26) | 1.000^b^ | -0.263 (26) | 1.000^b^ |
| MMSE | 0.000 (26) | 1.000^b^ | -0.037 (26) | 1.000^b^ | -0.029 (26) | 1.000^b^ | -0.198 (26) | 1.000^b^ | 0.022 (26) | 1.000^b^ |
| FRSP | -0.224 (27) | 1.000^b^ | 0.187 (27) | 1.000^b^ | 0.194 (27) | 1.000^b^ | -0.158 (27) | 1.000^b^ | 0.142 (27) | 1.000^b^ |
| Pack Years | 0.117 (27) | 1.000^b^ | 0.484 (27) | 0.091^b^ | 0.452 (27) | 0.158^b^ | -0.209 (27) | 1.000^b^ | -0.077 (27) | 1.000^b^ |
| Exacerbation Frequency | 0.110 (27) | 1.000^b^ | 0.288 (27) | 1.000^b^ | 0.306 (27) | 1.000^b^ | -0.020 (27) | 1.000^b^ | 0.140 (27) | 1.000^b^ |
| FEV_1_ (% pred.) | 0.135 (27) | 1.000^b^ | -0.015 (27) | 1.000^b^ | 0.019 (27) | 1.000^b^ | 0.054 (27) | 1.000^b^ | -0.137 (27) | 1.000^b^ |
| FVC (% pred.) | 0.260 (27) | 1.000^b^ | 0.305 (27) | 1.000^b^ | 0.322 (27) | 1.000^b^ | -0.193 (27) | 1.000^b^ | 0.045 (27) | 1.000^b^ |
| PO_2_ | -0.095 (27) | 1.000^b^ | -0.073 (27) | 1.000^b^ | -0.114 (27) | 1.000^b^ | 0.060 (27) | 1.000^b^ | -0.170 (27) | 1.000^b^ |
| PCO_2_ | -0.049 (27) | 1.000^b^ | -0.063 (27) | 1.000^b^ | -0.029 (27) | 1.000^b^ | -0.186 (27) | 1.000^b^ | 0.261 (27) | 1.000^b^ |
| SGRQ | -0.067 (27) | 1.000^b^ | 0.246 (27) | 1.000^b^ | 0.222 (27) | 1.000^b^ | 0.127 (27) | 1.000^b^ | 0.039 (27) | 1.000^b^ |

Age and sex were entered as confounders in all analyses. Additionally, estimated pre-morbid IQ was included in correlations involving cognitive function. Spearman’s correlation coefficients (*rho*), degrees of freedom (*df*) and *p*-values (*p*) are displayed. ^b^Bonferroni corrected *p*-values.
