# Supplementary material for: Disruption of white matter connectivity in chronic obstructive pulmonary disease
Source: PLoS One. 2019 Oct 3;14(10):e0223297. doi: 10.1371/journal.pone.0223297 (PMC6776415; doi:10.1371/journal.pone.0223297)
Supplement: S6 Table — Age and sex were entered as confounders in all analyses. Additionally, estimated pre-morbid IQ was included in correlations involving cognitive function. Spearman’s correlation coefficients (rho), degrees of freedom (df) and p-values (p) are displayed. bBonferroni corrected p-values. (DOCX) [file pone.0223297.s006.docx]

**S6 Table. Within-group correlations between global unweighted network metrics and cognitive and disease severity measures for the volume-adjusted weighting strategy**

| **Unweighted Global Network Metrics** | | | | | | | | | | |
| --- | --- | --- | --- | --- | --- | --- | --- | --- | --- | --- |
|  | **Degree** | | **Global Efficiency** | | **Local Efficiency** | | **Betweenness Centrality** | | **Small-worldness** | |
| **Controls (N=23)** | *rho (df)* | *p* | *rho (df)* | *p* | *rho (df)* | *p* | *rho (df)* | *p* | *rho (df)* | *p* |
| Executive Function | 0.582 (19) | 0.070^b^ | -0.295 (19) | 1.000^b^ | 0.173 (19) | 1.000^b^ | 0.481 (19) | 0.316^b^ | 0.025 (19) | 1.000^b^ |
| Episodic Memory | 0.164 (19) | 1.000^b^ | 0.250 (19) | 1.000^b^ | -0.033 (19) | 1.000^b^ | 0.008 (19) | 1.000^b^ | 0.243 (19) | 1.000^b^ |
| Processing Speed | 0.497 (19) | 0.257^b^ | 0.078 (19) | 1.000^b^ | 0.077 (19) | 1.000^b^ | 0.181 (19) | 1.000^b^ | 0.044 (19) | 1.000^b^ |
| Working Memory | 0.433 (19) | 0.564^b^ | -0.196 (19) | 1.000^b^ | -0.178 (19) | 1.000^b^ | -0.181 (19) | 1.000^b^ | -0.289 (19) | 1.000^b^ |
| MMSE | -0.072 (19) | 1.000^b^ | -0.159 (19) | 1.000^b^ | -0.039 (19) | 1.000^b^ | -0.015 (19) | 1.000^b^ | 0.270 (19) | 1.000^b^ |
| **COPD Patients (N=30)** | | | | | | | | | | |
| Executive Function | -0.053 (26) | 1.000^b^ | 0.395 (26) | 0.413^b^ | -0.341 (26) | 0.812^b^ | -0.017 (26) | 1.000^b^ | -0.220 (26) | 1.000^b^ |
| Episodic Memory | 0.275 (26) | 1.000^b^ | 0.189 (26) | 1.000^b^ | -0.274 (26) | 1.000^b^ | -0.447 (26) | 0.195^b^ | -0.384 (26) | 0.479^b^ |
| Processing Speed | 0.345 (26) | 0.781^b^ | 0.200 (26) | 1.000^b^ | -0.437 (26) | 0.226^b^ | -0.226 (26) | 1.000^b^ | -0.217 (26) | 1.000^b^ |
| Working Memory | 0.271 (26) | 1.000^b^ | 0.197 (26) | 1.000^b^ | -0.230 (26) | 1.000^b^ | -0.292 (26) | 1.000^b^ | -0.312 (26) | 1.000^b^ |
| MMSE | -0.008 (26) | 1.000^b^ | 0.208 (26) | 1.000^b^ | 0.049 (26) | 1.000^b^ | -0.150 (26) | 1.000^b^ | 0.046 (26) | 1.000^b^ |
| FRSP | -0.234 (27) | 1.000^b^ | -0.032 (27) | 1.000^b^ | 0.206 (27) | 1.000^b^ | -0.050 (27) | 1.000^b^ | 0.131 (27) | 1.000^b^ |
| Pack Years | 0.093 (27) | 1.000^b^ | 0.210 (27) | 1.000^b^ | 0.006 (27) | 1.000^b^ | -0.131 (27) | 1.000^b^ | 0.003 (27) | 1.000^b^ |
| Exacerbation Frequency | 0.115 (27) | 1.000^b^ | -0.043 (27) | 1.000^b^ | 0.013 (27) | 1.000^b^ | 0.232 (27) | 1.000^b^ | 0.230 (27) | 1.000^b^ |
| FEV_1_ (% pred.) | 0.127 (27) | 1.000^b^ | -0.314 (27) | 1.000^b^ | 0.132 (27) | 1.000^b^ | 0.026 (27) | 1.000^b^ | -0.112 (27) | 1.000^b^ |
| FVC (% pred.) | 0.243 (27) | 1.000^b^ | -0.264 (27) | 1.000^b^ | 0.233 (27) | 1.000^b^ | -0.128 (27) | 1.000^b^ | 0.066 (27) | 1.000^b^ |
| PO_2_ | -0.072 (27) | 1.000^b^ | 0.107 (27) | 1.000^b^ | -0.089 (27) | 1.000^b^ | 0.007 (27) | 1.000^b^ | -0.235 (27) | 1.000^b^ |
| PCO_2_ | -0.038 (27) | 1.000^b^ | 0.184 (27) | 1.000^b^ | 0.108 (27) | 1.000^b^ | -0.165 (27) | 1.000^b^ | 0.159 (27) | 1.000^b^ |
| SGRQ | -0.031 (27) | 1.000^b^ | 0.053 (27) | 1.000^b^ | 0.100 (27) | 1.000^b^ | 0.117 (27) | 1.000^b^ | 0.102 (27) | 1.000^b^ |

Age and sex were entered as confounders in all analyses. Additionally, estimated pre-morbid IQ was included in correlations involving cognitive function. Spearman’s correlation coefficients (*rho*), degrees of freedom (*df*) and p-values (*p*) are displayed. ^b^Bonferroni corrected *p*-values. *significant at *p*<0.05.
