# Supplementary figures and images for: Disruption of white matter connectivity in chronic obstructive pulmonary disease
Source: PLoS One. 2019 Oct 3;14(10):e0223297. doi: 10.1371/journal.pone.0223297 (PMC6776415; doi:10.1371/journal.pone.0223297)

## Unweighted

## Weighted

### Integration

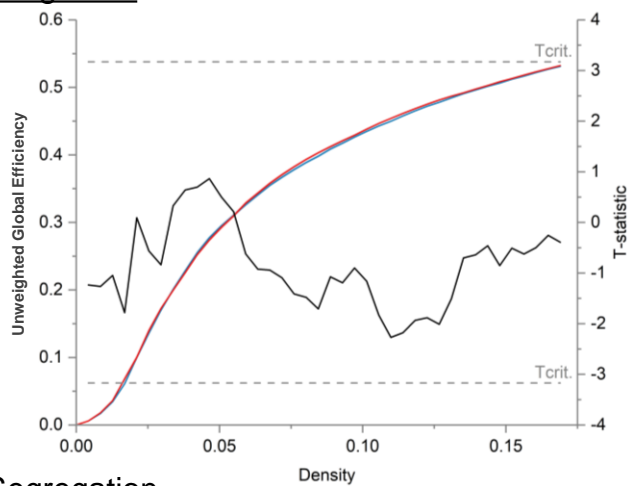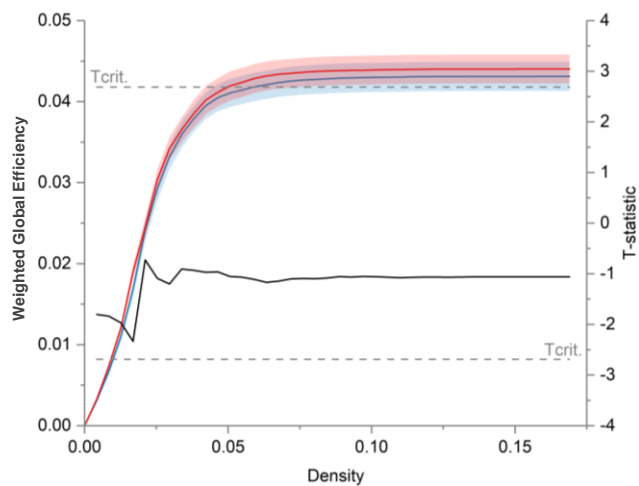

### Segregation

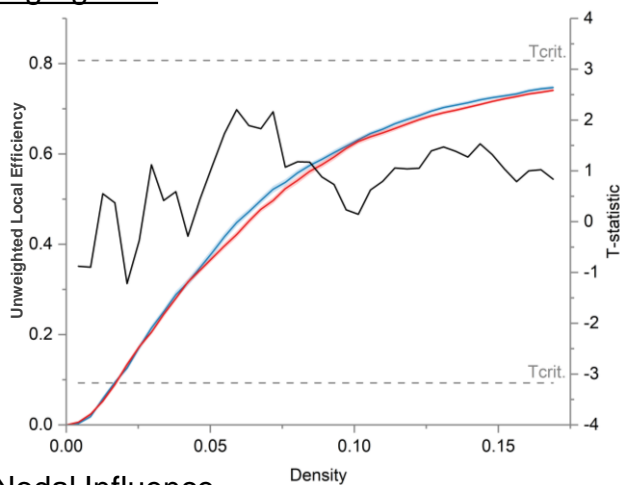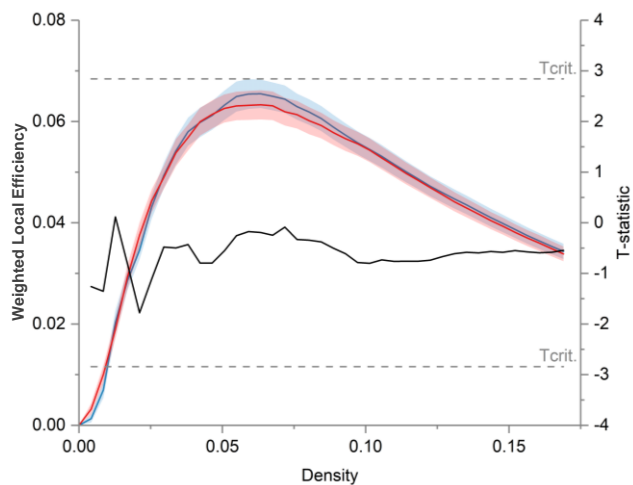

### Nodal Influence

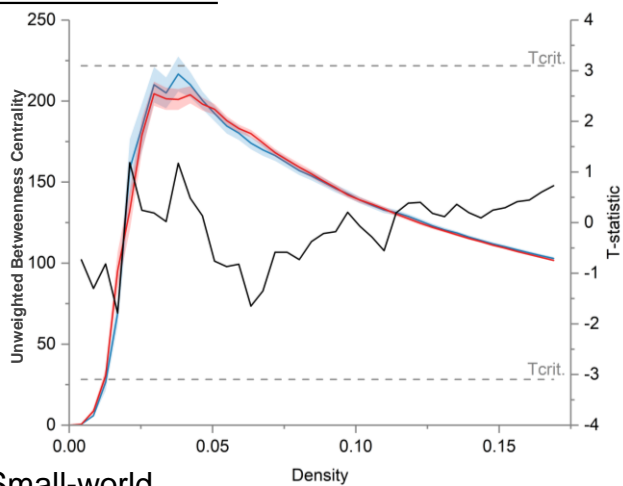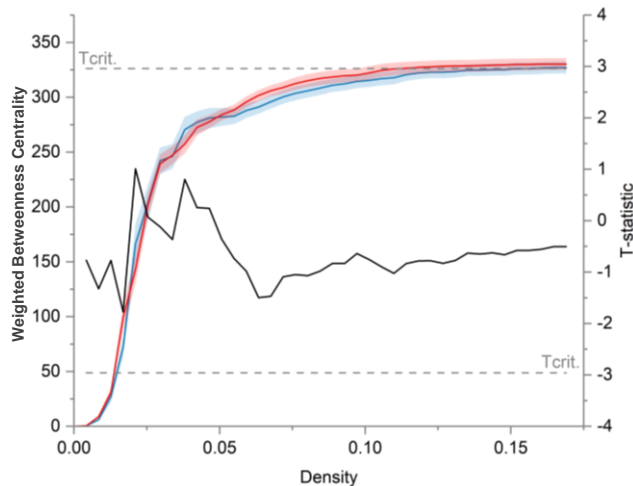

### Small-world

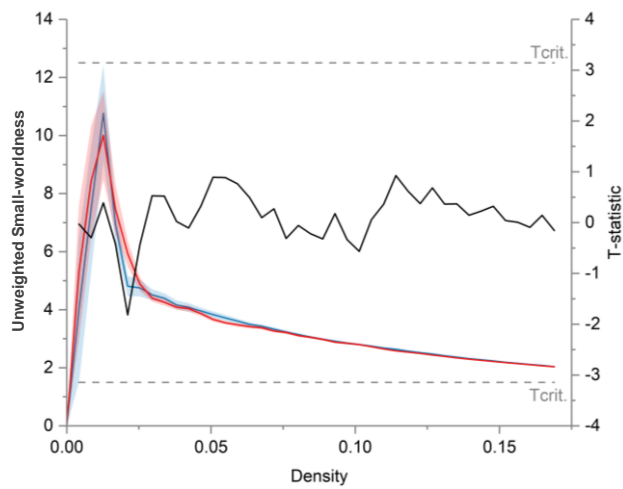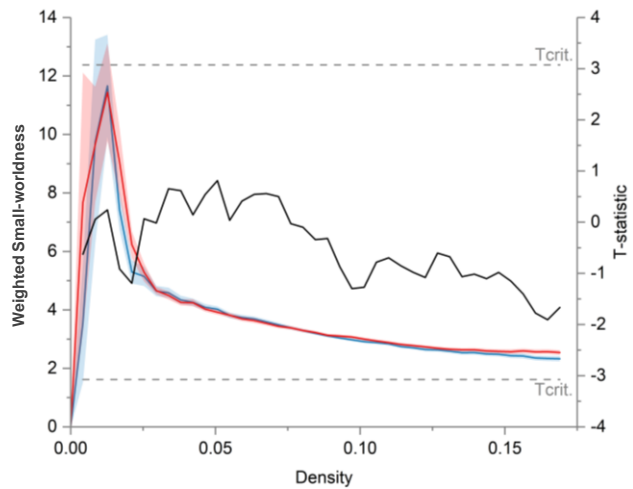

Supplement: S1 Fig — Group average metric curves for unweighted and weighted global network metrics are plotted on the left axes. Red = COPD patients, Blue = Controls. Shaded error bars represent the standard error of the mean. T-statistics (black) are plotted on the right axis. Two-tailed critical thresholds (Tcrit) are indicated by dashed grey lines. (PDF) [file pone.0223297.s007.pdf]

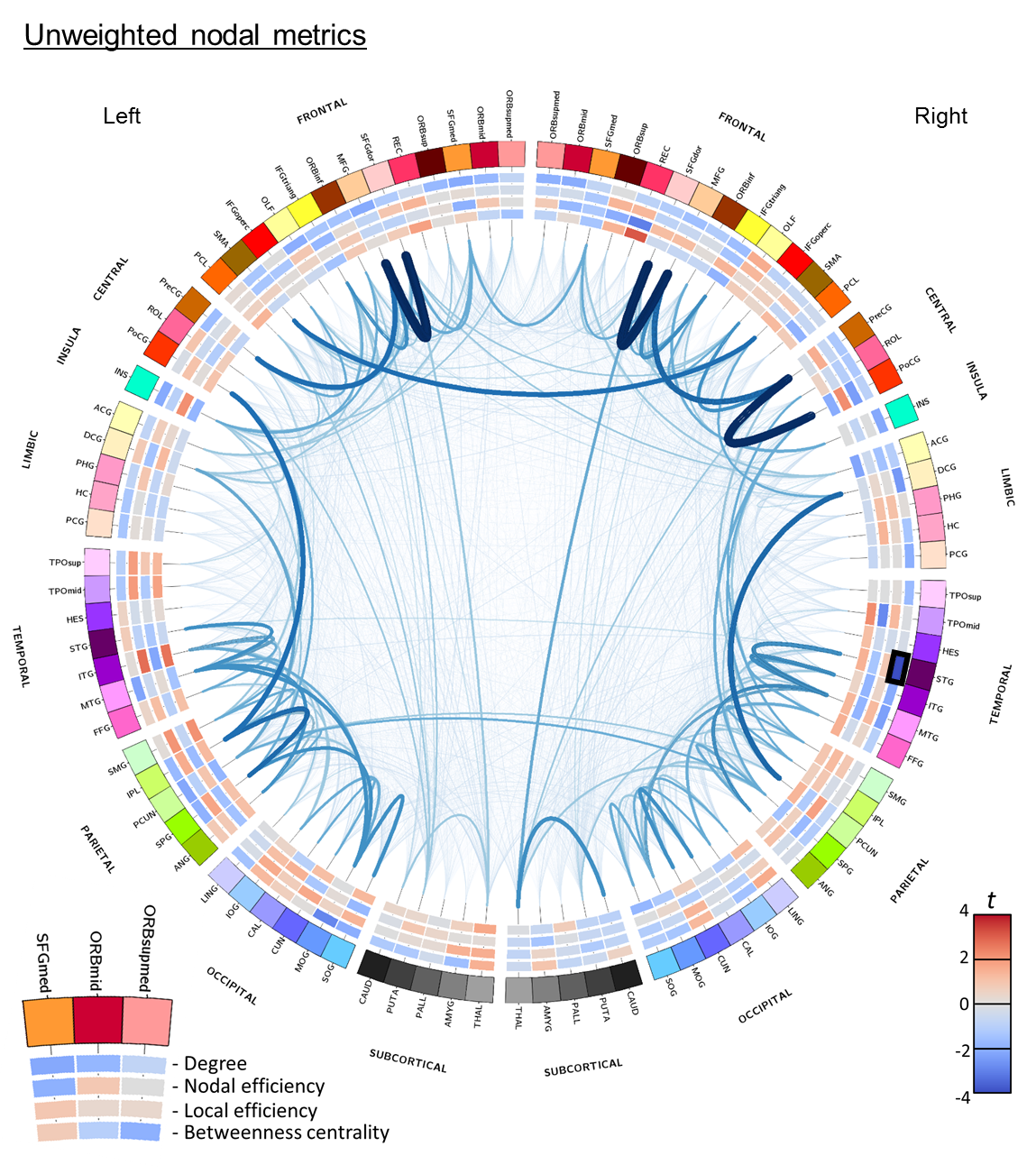

Supplement: S3 Fig — Network nodes are arranged around the outermost circle and assigned a unique colour. Nodes are split by hemisphere (right hemisphere on the right) and grouped within the macroscopic subdivisions defined in [32] (Frontal, Central, Insula, Limbic, Temporal, Parietal, Occipital, Subcortical). Within these subdivisions nodes are arranged by structural laterality. S3 Table summarises the node name abbreviations. The inner four circles show red-blue t-statistic heatmaps for between-group AUCtotal differences in nodal unweighted metrics for the contrast COPD patients>controls. Connections represent the edges present in any subject. The thickness and darkness of connections indicates the average edge weight for the streamline length-adjusted weighting strategy. Significant results are outlined in black. (TIF) [file pone.0223297.s009.tif]

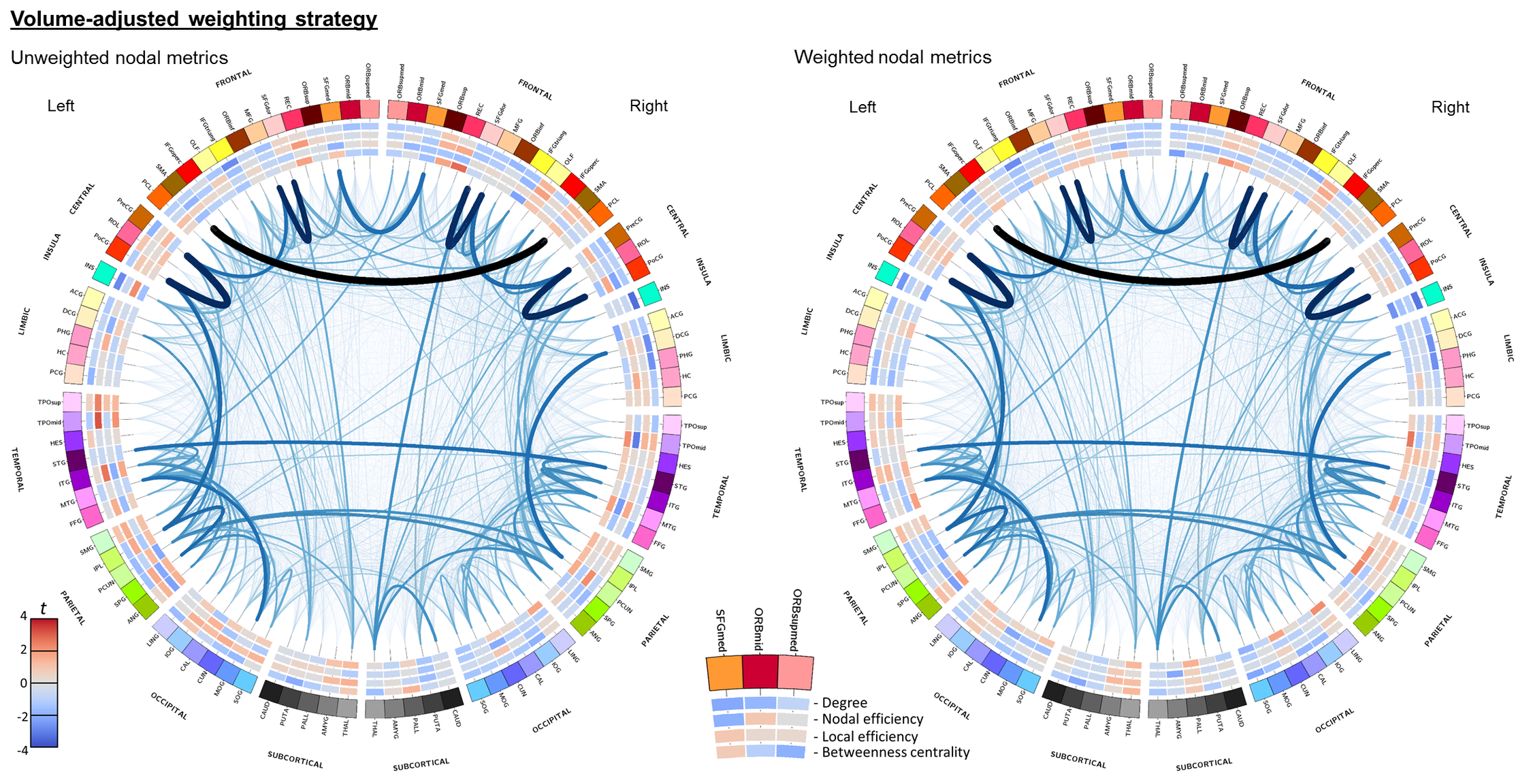

Supplement: S4 Fig — Network nodes are arranged around the outermost circle and assigned a unique colour. Nodes are split by hemisphere (right hemisphere on the right) and grouped within the macroscopic subdivisions defined in [32] (Frontal, Central, Insula, Limbic, Temporal, Parietal, Occipital, Subcortical). Within these subdivisions nodes are arranged by structural laterality. S3 Table summarises the node name abbreviations. The inner four circles show red-blue t-statistic heatmaps for the sub-significant between-group AUCtotal trends in nodal unweighted metrics for the contrast COPD patients>controls. Connections represent the edges present in any subject. The thickness and darkness of connections indicates the average edge weight for the volume-adjusted weighting strategy. (TIF) [file pone.0223297.s010.tif]
