# Supplementary material for: Disruption of white matter connectivity in chronic obstructive pulmonary disease
Source: PLoS One. 2019 Oct 3;14(10):e0223297. doi: 10.1371/journal.pone.0223297 (PMC6776415; doi:10.1371/journal.pone.0223297)
Supplement: S2 Fig — Group average metric curves for unweighted and weighted global network metrics are plotted on the left axes. Red = COPD patients, Blue = Controls. Shaded error bars represent the standard error of the mean. T-statistics (black) are plotted on the right axis. Two-tailed critical thresholds (Tcrit) are indicated by dashed grey lines. *significant at PFWE<0.05 after MTPC correction for multiplicity. (PDF) [file pone.0223297.s008.pdf]

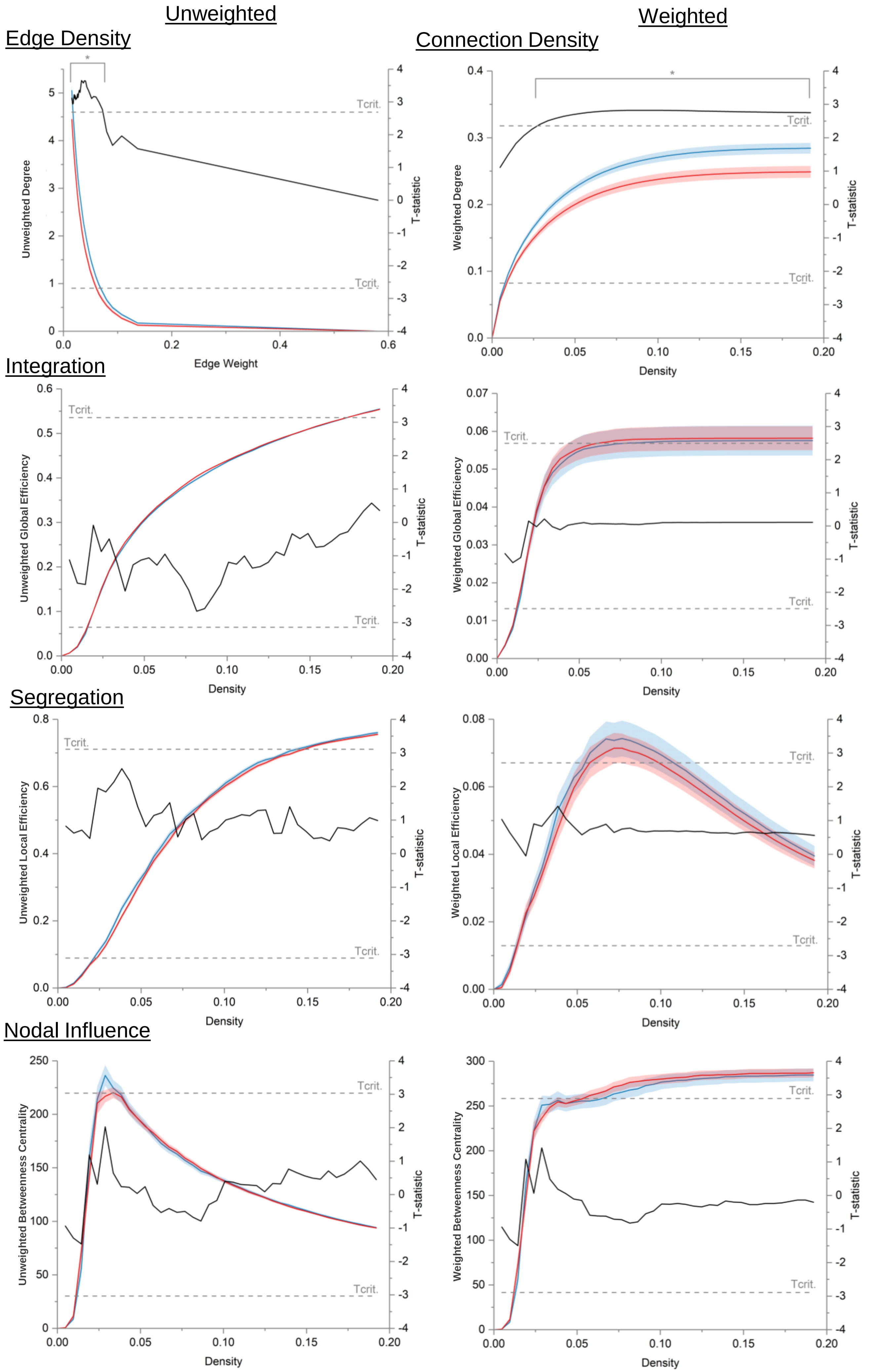
Unweighted
Weighted
Edge Density
Connection Density
Unweighted Degree
Weighted Degree
Edge Weight
Density
T-statistic
Tcrit.
*
Integration
Unweighted Global Efficiency
Weighted Global Efficiency
Segregation
Unweighted Local Efficiency
Weighted Local Efficiency
Nodal Influence
Unweighted Betweenness Centrality
Weighted Betweenness Centrality

Small-world

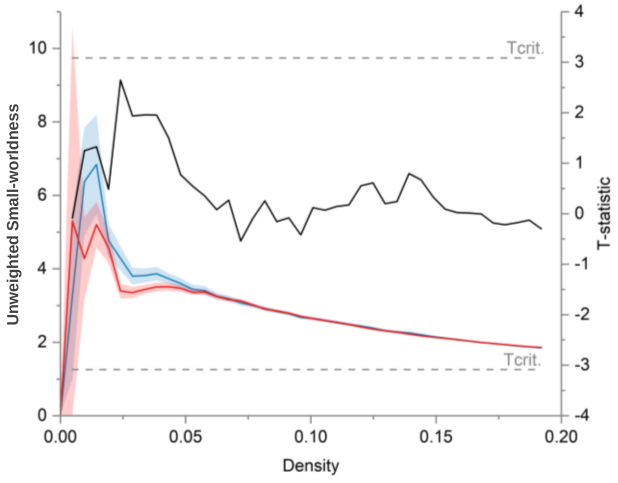

Unweighted Small-worldness
T-statistic
Density
Tcrit.
Tcrit.

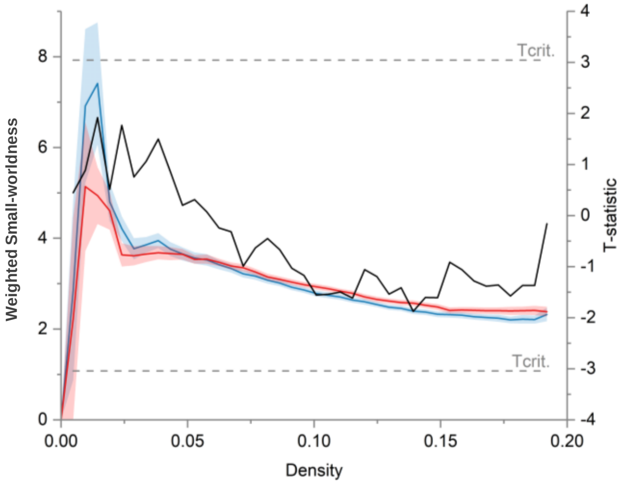

Weighted Small-worldness
T-statistic
Density
Tcrit.
Tcrit.
