# Supplementary material for: Disruption of white matter connectivity in chronic obstructive pulmonary disease
Source: PLoS One. 2019 Oct 3;14(10):e0223297. doi: 10.1371/journal.pone.0223297 (PMC6776415; doi:10.1371/journal.pone.0223297)
Supplement: S2 Table — Point-by-point group comparison of global network measures. Age and sex were included as confounders in all analyses. For the maximum statistical difference (Peak), the t-statistic (tmax), degrees of freedom (df), permutation-based family-wise error corrected p-value (pFWE) and the network threshold at which this difference occurs (τ) are reported under the heading ‘Peak statistics’. Additionally, the size of supra-critical clusters (AUCMTPC), the critical threshold for these clusters (AUCcrit) and their significance (MTPCsig), Y = yes, N = no are reported under the heading ‘Cluster’. (DOCX) [file pone.0223297.s002.docx]

**S2 Table. Group comparison of global network metrics for the volume-adjusted weighting strategy – ‘point-by-point’ along the metric curve**

|  | **Peak statistics** | | | **Cluster** | | |
| --- | --- | --- | --- | --- | --- | --- |
| **Unweighted Network Metrics** | ***t*_max_ *(df)*** | ***p*_FWE_** | ***τ*** | **AUC_MTPC_** | **AUC_crit_** | **MTPC_sig_** |
| Degree | 3.653 (49) | 0.005 | 0.033 | 0.034 | 0.010 | Y |
| Global Efficiency | -2.660 (49) | 0.159 | 0.110 | - | 0.003 | N |
| Local Efficiency | 2.531 (49) | 0.175 | 0.154 | - | 0.004 | N |
| Betweenness Centrality | 2.027 (49) | 0.410 | 0.163 | - | 0.005 | N |
| Small-worldness | 2.648 (49) | 0.131 | 0.024 | - | 0.004 | N |
| **Weighted Network Metrics** |  |  |  |  |  |  |
| Degree | 2.822 (49) | 0.018 | 0.101 | 0.066 | 0.039 | Y |
| Global Efficiency | -1.107 (49) | 0.543 | 0.182 | - | 0.024 | N |
| Local Efficiency | 1.424 (49) | 0.613 | 0.154 | - | 0.013 | N |
| Betweenness Centrality | -1.495 (49) | 0.707 | 0.178 | - | 0.008 | N |
| Small-worldness | 1.918 (49) | 0.497 | 0.014 | - | 0.005 | N |

Point-by-point group comparison of global network measures. Age and sex were included as confounders in all analyses. For the maximum statistical difference (Peak), the *t*-statistic (*t_max_*), degrees of freedom (*df*), permutation-based family-wise error corrected *p-*value (*p_FWE_*) and the network threshold at which this difference occurs (*τ*) are reported under the heading ‘Peak statistics’. Additionally, the size of supra-critical clusters (AUC_MTPC_), the critical threshold for these clusters (AUC_crit_) and their significance (MTPC_sig_), Y=yes, N=no are reported under the heading ‘Cluster’.
